# Genetic Dissection of ToLCNDV Resistance in Resistant Sources of *Cucumis melo*

**DOI:** 10.3390/ijms25168880

**Published:** 2024-08-15

**Authors:** Clara Pérez-Moro, Cristina Sáez, Alicia Sifres, Carmelo López, Narinder P. S. Dhillon, Belén Picó, Ana Pérez-de-Castro

**Affiliations:** 1 Instituto de Conservación y Mejora de la Agrodiversidad Valenciana (COMAV-UPV), Universitat Politècnica de València, Camino de Vera s/n, 46022 Valencia, Spain; clapemo@alumni.upv.es (C.P.-M.); cristina.saez@upm.es (C.S.); alsifcue@upvnet.upv.es (A.S.); clopez@upv.es (C.L.); 2 Centro de Biotecnología y Genómica de Plantas UPM-INIA and E.T.S. Ingeniería Agronómica, Alimentaria y de Biosistemas, Universidad Politécnica de Madrid, 28031 Madrid, Spain; 3 World Vegetable Center, East and Southeast Asia, Kasetsart University, Kamphaeng Saen, Nakhon Pathom 73140, Thailand; narinder.dhillon@worldveg.org

**Keywords:** melon, *Cucumis melo*, ToLCNDV, BSR-seq, virus resistance, QTL, gene expression

## Abstract

Tomato leaf curl New Delhi virus (ToLCNDV) is a begomovirus causing significant melon (*Cucumis melo*) crop losses globally. This study aims to map the ToLCNDV resistance in the PI 414723 melon accession, previously identified and characterized through phenotypic studies, thereby exploring shared genomic regions with the established resistant source WM-7. In the present study, WM-7 and PI 414723 were crossed with the susceptible accessions ‘Rochet’ and ‘Blanco’ respectively, to generate F_1_ hybrids. These hybrids were self-pollinated to generate the populations for mapping the ToLCNDV resistance region and designing markers for marker-assisted selection. Disease evaluation included visual symptom scoring, viral-load quantification and tissue printing. Genotyping-by-sequencing and SNP markers were used for quantitative trait loci (QTL) mapping. For genetic analysis, qPCR and bulked segregant RNA-seq (BSR-seq) were performed. Gene expression was assessed using RNA-seq, and qRT-PCR was used for confirmation. The research narrows the candidate region for resistance in WM-7 and identifies overlapping QTLs on chromosome 11 in PI 414723, found in the region of the DNA primase large subunit. BSR-seq and expression analyses highlight potential regulatory roles of chromosome 2 in conferring resistance. Differential expression was confirmed for six genes in the candidate region on chromosome 2. This study confirms the existence of common resistance genes in PI 414723 and WM-7.

## 1. Introduction

Tomato leaf curl New Delhi virus (ToLCNDV) is a bipartite *Begomovirus* naturally transmitted by the whitefly *Bemisia tabaci* in a persistent manner. ToLCNDV was first reported in India on tomato (*Solanum lycopersicum* L.) [1,2] and subsequently spread throughout other Asian countries [3]. In 2012, it was detected in zucchini (*Cucurbita pepo* L.) crops of Murcia greenhouses and was reported shortly after in Almeria [4]. ToLCNDV has been reported in Asia (Bangladesh, China, India, Indonesia, Iran, Nepal, Pakistan, Philippines, Sri Lanka, Taiwan and Thailand), Europe (Estonia, France, Greece, Italy, Lithuania, Portugal, Slovakia, Slovenia and Spain) and Africa (Algeria, Morocco, Seychelles and Tunisia) [5]. The Spanish isolates of ToLCNDV have been considered a new strain, designated as the Mediterranean strain (ToLCNDV-ES), which is better adapted to infect cucurbits [6].

ToLCNDV epidemics cause severe yield losses in economically important crop families such as *Solanaceae* and *Cucurbitaceae*. Their symptomatology includes severe mosaic, leaf curl, vein thickening and puckering of the leaves [7]. In melon crops (*Cucumis melo* L.), ToLCNDV infections at early developmental stages can cause a reduction in plant growth and flowering, with skin roughness in fruits, longitudinal cracking and small fruit size, thus decreasing fruit quality, to the point of rendering them unmarketable [4,7].

Melon is an economically important horticultural crop. In 2022, world melon production reached 28.6 million tons and covered a cultivated area of 1.06 million hectares [8]. China was the main producer, with a total 14 million tons. In Spain, melon production accounts for more than 16,220 ha in greenhouse and open-field environments, with production of 524,040 tons in 2022, representing one third of total European production [8]. Melon is cultivated throughout the country, with the Andalucía and Murcia regions representing about 60% of total Spanish production [9]. The presence of severe viral diseases such as ToLCNDV undermines melon cultivation.

In the absence of genetic resistance, the main strategies employed to control ToLCNDV are those based on the control of whiteflies, such as the application of insecticides, with consequent environmental risk and the threat of vectors developing resistance to said insecticides. A more effective method of reducing the spread of ToLCNDV is the integrated whitefly management approach, based on biological control with *Amblyseius swirskii.* However, its application is currently limited in scale [10]. Other cultural practices such as the managing of planting dates, rotation systems and crop-free periods are used, with the disadvantage of requiring a high degree of local coordination between growers. In contrast, host genetic resistance is considered highly effective in defense against viral infection in the field. Genetic resistance, combined with cultural practices, could become a viable way to reduce the economic losses produced by virus infection [11].

The first cucurbit reported as resistant to ToLCNDV was sponge gourd (*Luffa cylindrica* M. Roem), displaying monogenic-dominant resistance [12]. Currently, at least 18 resistant/tolerant melon accessions have been identified in multiple germplasm screenings (reviewed in [13,14]). López et al. [15] and Sáez et al. [16] identified different levels of resistance to ToLCNDV in PI 124112, PI 414723 and Kharbuja from the momordica melon group, as well as in the wild accessions WM-7 and WM-9 belonging to the kachri melon group. Notably, all of the five accessions came from India, the country where this virus was originally reported and where the highest variability in melon has been reported, being considered as the main center of melon diversity [17]. In areas where the virus has persisted over time, plants have had a longer evolutionary time to develop resistance, making the identification of sources of resistance in such areas more likely.

WM-7-derived resistance has been reported as conferred by a main *locus* located on chromosome 11 and in two regions of minor effect on chromosomes 2 and 12 [16]. When the alleles of the resistance *locus* of chromosome 11 were in homozygosis, inoculated plants remained asymptomatic. However, when this *locus* was heterozygous, the regions of chromosomes 2 and 12 modulated symptom display and virus titer accumulation. Similar results were reported for the IC-274014 melon accession (momordica group), with one recessive gene and two dominant genes controlling ToLCNDV-ES resistance [18].

Sáez et al. [19] compared gene expression changes between WM-7 and Piel de sapo (a Spanish variety, belonging to the ibericus melon group, highly susceptible to ToLCNDV) melon plants after ToLCNDV inoculation. In this study, the gene encoding a DNA primase large subunit (MELO3C022319.2), located within the candidate region for ToLCNDV resistance on chromosome 11, was found to be down-regulated in resistant plants. Moreover, the orthologue gene of MELO3C022319.2 in *Cucurbita moschata* (Duchesne) (CmoCh08G001720) is located within the main candidate region for ToLCNDV resistance on chromosome 8 of this species [20]. These findings pointed to a DNA primase large subunit as the main candidate resistance gene for resistance to ToLCNDV. Later, in silico analysis identified a non-synonymous mutation (position 30,355,908 bp in *C. melo* genome v.4.0) affecting this gene on the genomes of different resistant melon accessions, which was associated with ToLCNDV resistance [21]. Furthermore, silencing the orthologue of MELO3C022319.2 in *Nicotiana benthamiana* caused a reduction in virus titer after infection with different geminiviruses, validating the role of this gene in geminiviral replication [21].

Resistance to ToLCNDV in melon was also identified in the accession PI 414723 [15]. Most plants of PI 414723 remained symptomless after ToLCNDV inoculation, and although a few showed moderate symptoms at 15 days post inoculation (dpi), all recovered and were symptomless by the end of the assay [16]. Furthermore, PI 414723 has been reported as resistant to zucchini yellow mosaic virus (ZYMV); this resistance has been explained by both the monogenic-dominant resistance model (*Zymv*) and the three-dominant-complementary-genes model (*Zym-1, Zym-2, Zym-3*) [22,23]. Additionally, PI 414723 has shown resistance to powdery mildew caused by *Podosphaera xanthii*, papaya ring-spot virus (PRSV), watermelon mosaic virus (WMV), cucurbit aphid-borne yellow virus (CABYV) and *Aphis gossypii*. PI 414723 was collected in India, similarly to ToLCNDV-resistant accessions, such as WM-7. This may be related to host–pathogen co-evolution in this area, as it was in India that the infection was first detected [15].

In this study, we advance the dissection of genetic resistance to ToLCNDV by narrowing the chromosome 11 candidate region from WM-7 and validating the resistance in an ibericus genetic background other than the ‘Piel de Sapo’. Furthermore, we studied the genetic control of resistance to ToLCNDV derived from the multiresistant source PI 414723. Segregating populations were used to map the QTLs associated with virus resistance, and BSR-seq analysis allowed for the identification of additional genomic regions and candidate genes. The results from the present study will aid and strengthen melon-breeding programs for ToLCNDV resistance, providing SNP markers useful to select and introgress resistance alleles coming from different melon sources.

## 2. Results

### 2.1. Narrow Down Chromosome 11 Candidate Region for WM-7-Derived ToLCNDV Resistance

#### 2.1.1. Genotyping-by-Sequencing

The ToLCNDV-resistant source WM-7 was crossed with a homozygous line derived from the Spanish melon landrace ‘Rochet’ (RC-BGV003718), highly susceptible to ToLCNDV. F_1_ generation was selfed to produce the F_2_ generation. Plants from this segregating population were genotyped with a set of 24 markers (Table S1), covering the three genomic regions associated with the ToLCNDV of chromosomes 2, 11 and 12 [16]. One of the plants, homozygous for WM-7 alleles in the candidate region of chromosome 2 and homozygous for the RC allele in the candidate region of chromosome 12, was selected (Table 1). This plant was heterozygous between SNPCmND7 (30,249,798 bp) and SNPCmND13bis (30,347,863 bp) in the candidate region of chromosome 11 and homozygous for the RC allele for the rest of the SNPs in this region (Table 1). This plant was selfed and the F_3_ offspring obtained were phenotyped for resistance to ToLCNDV, showing segregation for resistance. This information allowed us to narrow down the candidate region for ToLCNDV resistance established in previous works [16], setting the new end limit at 30,377,414 bp.

The five most susceptible (numbered 11, 17, 14, 7 and 4b) and the two fully resistant (numbered 8 and 4) F_3_ plants were included in a genotyping-by-sequencing (GBS) assay, along with WM-7 and RC parents.

The sequencing of the nine plants resulted in an average of 6.1 million raw reads (Table S2). The average mapping rate of the trimmed and filtered reads ranged from 96% to 97%. A variant-calling procedure was performed, obtaining 54,384 SNPs after filtering. The SnpEff program revealed that 159 SNPs presented a high impact (e.g., those affecting splice sites, start and stop codons), 1411 SNPs presented a moderate impact (e.g., non-synonymous variations), 1501 SNPs presented a low impact (e.g., synonymous variations in coding regions and start and stop codons) and 51,313 were modifiers (variations in non-coding regions: upstream, downstream, intergenic and UTR regions) (Table S2).

Thirty-two GBS-derived SNPs were identified within the segregating candidate region on chromosome 11 (Table 2). The genotype of the susceptible-to-ToLCNDV F_3_ plant “14” allowed us to set the upper limit in position 30,159,412 bp, as the plant is homozygous for the WM-7 allele in this position. All the plants were found to be homozygous for WM-7 alleles in the candidate region of chromosome 2 (20,359,078–25,729,272 bp) and homozygous for the RC allele for the candidate region on chromosome 12 (0–20,966,749 bp) [16] for those markers homozygous in the corresponding parentals (Table S2).

Based on these results, the region linked to ToLCNDV resistance was established between 30,159,412 and 30,377,414 bp of the melon chromosome 11, with these positions corresponding to marker S11_30159412 (Table 2) and marker SNPCmND15 (Table 1), reducing the candidate region by 218 Kb compared to the previous interval (30,112,560–30,737,924 bp) [16,19]. Among these detected SNPs, both S11_30358926 (T/G) and S11_30359039 (A/T) had a modifier effect on MELO3C022319.2, coding a DNA primase large subunit, and both are located downstream of MELO3C022319.2. The results of the GBS made it possible to design new markers saturating the target and flanking regions of MELO3C022319.2 (physical position 30,347,186–30,355,934 bp) between WM-7 and RC, useful for marker-assisted selection (MAS) with this group of commercially interesting melons (Material and Methods, Section 4.3).

Three high-impact variants were found in the candidate region of chromosome 2 [16], with two affecting MELO3C017345.2, which encodes detoxification protein, and one affecting MELO3C017328.2 (stress up-regulated Nod 19 protein). Five high-effect variants, polymorphic among the resistant and the susceptible parentals, were found in the candidate region of chromosome 12 [16], with one affecting MELO3C004853.2, which encodes 30S ribosomal protein S3, and the other four affecting MELO3C035485.2 (gamma-aminobutyrate transaminase POP2, mitochondrial-like), MELO3C035514.2 (Unknown protein), MELO3C035533.2 (gamma-aminobutyrate transaminase POP2, mitochondrial-like) and MELO3C025574.2 (flocculation protein FLO11-like). None of the seven genes were differentially expressed between WM-7 and PS after ToLCNDV infection [19].

#### 2.1.2. Cosegregation Analysis in the F_3_ Population

To confirm the effect of the candidate region of chromosome 11, all plants of the F_3_ population previously mentioned were classified according to their genotype for marker SNPCmND13bis (Table 1), located at 30,347,863bp in chromosome 11, and markers NDHRM08 and NDHRM26 (SNPs S11_30339408 and S11_30358926, respectively), detected in the GBS (Table 2) and converted into High Resolution Melting (HRM) markers (Table S3). Recombination was not detected between the markers.

Differences in symptom score at 15 dpi and 30 dpi were significant between genotypic classes (*p* = 0.0036 and *p* < 0.001 respectively) (Figure S1). In addition, relative ToLCNDV accumulation was significantly different between genotypic classes at 15 dpi (*p* = 0.0088) and 30 dpi (*p* = 0.0022).

All the F_3_ plants homozygous for the WM-7 allele exhibited mild symptoms or were asymptomatic, and the average virus titer was lower than in the homozygous for the RC allele and heterozygous F_3_ plants.

Symptom variability, from 0 to 4 in symptom score, was found in plants that were homozygous for the RC allele and heterozygous (Figure S1).

**Figure 1:**
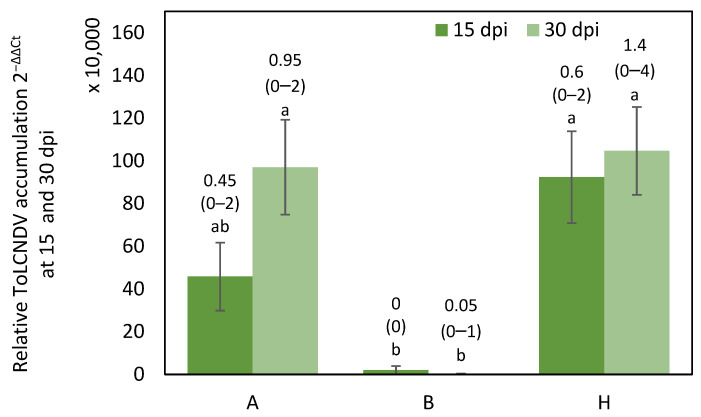
Average relative ToLCNDV accumulation (calculated as 2^−ΔΔCt^) at 15 dpi (days post inoculation) and 30 dpi in F_3_ plants derived from the initial cross WM-7 × RC. Genotypes are classified according to the genotype for the three cosegregating markers SNPCmND13bis, NDHRM26 and NDHRM08, where A is homozygous for RC allele, H is heterozygous and B is homozygous for WM-7 allele. Bars represent standard error. Different letters indicate significant differences among genotypes at the same date (*p* < 0.05, LSD test). Average and range of symptoms are shown above the bars.

No significant differences were found in viral titer between heterozygous (H) and RC homozygous (A) plants for the three markers (Figure 1), but significant differences were found in symptoms at 30 dpi between A and H (*p* = 0.0196) (Figure S1), despite this F_3_ population being homozygous for the WM-7 alleles in the candidate region of chromosome 2 that includes the marker CMPSNP658 (25,174,774). Our findings differ from those reported by Sáez et al. [16]. In their study, which employed a different population derived from the cross WM-7 × PS ‘Piñonet’, with an ibericus “Piel de Sapo” background, they observed an epistatic effect involving the chromosome 2 and 11 regions.

### 2.2. Fine Mapping of PI 414723-Derived Resistance

#### 2.2.1. Phenotypic Variation in ToLCNDV Resistance in the F_2_ Population

**Figure 2:**
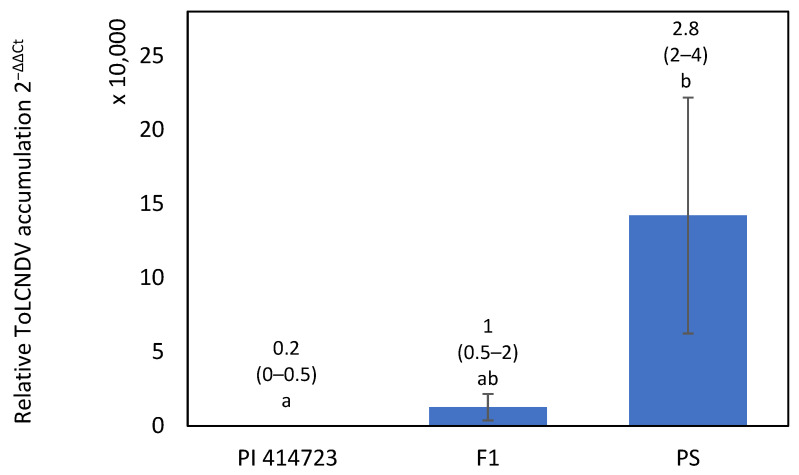
Relative ToLCNDV accumulation (calculated as 2^−ΔΔCt^) at 30 dpi with ToLCNDV in PI 414723, Piel de Sapo (PS ‘Piñonet’) and F_1_ derived from the cross PI 414723 × Piel de Sapo (PS ‘Piñonet’). Bars represent standard error. Different letters indicate significant differences among genotypes at the same date (*p* < 0.05, LSD test). The average and range of symptoms are shown above the bars.

The resistant response to ToLCNDV of PI 414723 was characterized in an inoculation assay including both parents and the F_1_, F_2_ and BC generations derived from the cross PI 414723 × Piel de Sapo (PS ’Piñonet’). Most plants of the resistant parent, PI 414723, remained asymptomatic, but some of them showed mild symptoms, scored as 1, at 15 dpi. All of them recovered and were symptomless at the end of the assay and accumulated low virus titer at 30 dpi (Figure 2). In contrast, all PS ‘Piñonet’ plants behaved as highly susceptible to the virus. The first symptoms appeared at 15 dpi and were scored 4, severe, at 30 dpi. All of them had high virus titers. Some F_1_ plants showed mild or moderate symptoms, scored as 2 at 15 dpi, and had moderate virus titers (Figure 2). Most of the plants had recovered from their symptoms by the end of the assay, except for two of them, which retained moderate symptoms.

In the F_2_ population at the end of the assay, 98 plants were classified as susceptible (scores 2 to 4), 59 plants were classified as resistant (scores 0 and 1) and 12 plants died for causes unrelated to ToLCNDV infection. BC_PS_ also segregated for symptom severity, with 9 resistant (scores 0 to 1) and 13 susceptible (scores 2 to 4) plants detected at 30 dpi. These results did not fit a simple genetic model, confirming the effect of several loci over ToLCNDV resistance in PI 414723 accession.

#### 2.2.2. QTL Analysis of F_2_ Population

A total of 169 plants from the F_2_ population (PI 414723 × PS ‘Piñonet’) were genotyped with a set of 124 SNP markers distributed throughout the melon genome, previously designed in different populations derived from crosses between occidental melons (ibericus or cantalupensis) and oriental melons (momordica, makuwa, acidulus, kakhri, etc.) [16,24,25,26,27,28]. Seventy-eight of them were polymorphic between the parental lines (Table S4) and used to construct a linkage map, which included 12 linkage groups, covering 924 cM with an average distance between consecutive markers of 27.8 cM. The linkage map was used in the analysis to identify QTLs involved in ToLCNDV resistance derived from PI 414723, based on genotyping results (Table S5), symptoms at 9, 15 and 30 dpi and virus titers at 30 dpi. Two QTLs were found, with both of them located in an overlapping region in chromosome 11: one for symptoms at 9 dpi and the other for virus titers at 30 dpi. No candidate QTLs were found associated with symptom score at 15 dpi and 30 dpi (Table 3) (Figure S2).

The CPMSNP315 marker, located at 30,194,725 bp on chromosome 11, was the closest marker to the LOD peak for both traits’ QTLs, ‘9 DPI’ and ‘30 DPI VT’, and explained 20.2% and 13.7% of the observed variation, respectively (Table 3). Both QTLs overlapped with the candidate region for ToLCNDV resistance derived from WM-7 (positions 30,159,412–30,377,414 bp on chromosome 11), including MELO3C022319.2.

#### 2.2.3. Validation of the Markers Linked to the WM-7 Candidate Region in PI 414723 F_2_ Populations with a Different Ibericus Genetic Background

As stated above, the QTL associated with PI 414723-derived resistance identified in chromosome 11 in the composite interval mapping (CIM) analysis overlapped with the candidate interval for WM-7-derived resistance and included the candidate gene, MELO3C022319.2 (DNA primase large subunit protein). The marker CPMSNP315, the closest marker to the QTLs peak associated with both traits, with symptoms at 9 dpi and virus titer at 30 dpi, is located at position 30,194,725 bp. In order to validate the new markers designed for MAS in WM-7-derived resistance in PI 414723-derived populations, one population derived from one additional ibericus genetic background, a traditional variety of the Blanco type (BL), was tested. The F_2_ population derived from the initial cross between PI 414723 and this variety was phenotyped for resistance and genotyped with the marker SNPCmND13bis (30,347,863) and the new marker derived from the GBS analysis, NDHRM26 (30,358,926), located near MELO3C022319.2.

**Figure 3:**
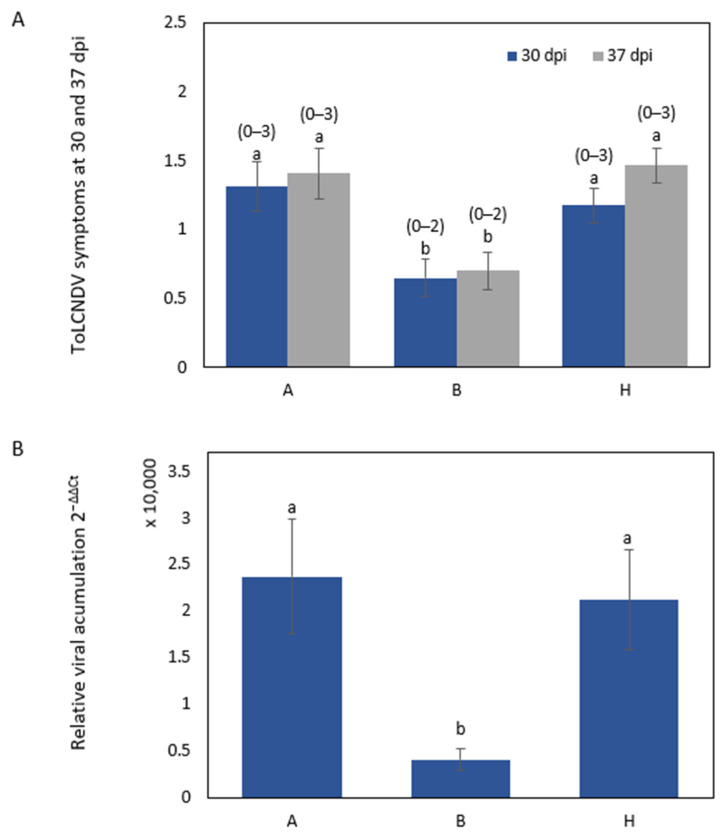
ToLCNDV inoculation response in F_2_ population plants derived from the cross PI 414723 × BL. Genotypes are classified according to the genotype for the marker NDHRM26, where A is homozygous for BL allele, H is heterozygous and B is homozygous for PI 414723 allele. (**A**) The average symptom score at 30 and 37 days post inoculation (dpi) with ToLCNDV in F_2_ population plants derived from the cross PI 414723 × BL, with the range of symptoms shown above the bars, and (**B**) relative ToLCNDV accumulation (calculated as 2^−ΔΔCt^) at 30 dpi in the F_2_ population derived from the cross PI 414723 × BL. Bars represent the standard error. Different letters indicate significant differences among genotypes on the same date (*p* < 0.05, LSD test).

Symptom scores significantly differed between plants classified according to the genotypes for the NDHRM26 marker at 30 dpi (*p* = 0.0061) and at 37 dpi (*p*= 0.0005) (Figure 3A); at both dates, the symptom score in the plants homozygous for the PI 414723 allele (B) was significantly different from those of the other genotypes. Similarly, the virus titer detected by quantitative polymerase chain reaction (qPCR) at 30 dpi significantly differed between plants homozygous for the PI 414723 allele and the other genotypes (*p* = 0.0213) (Figure 3B). The same results were found for the SNPCmND13bis marker.

In any case, the genotype for this region in chromosome 11 was not enough to explain the resistance. A total of 6 of 39 plants homozygous for the PI 414723 allele for the markers were scored with symptoms equal to or over 2. Specifically, four plants were scored as 2 and two were scored as 3 at the end of the assay. These results support the existence of additional regions implicated in the resistance derived from this source. The characterization of PI 414723-derived resistance in F_2_ generations in the two different genetic backgrounds (PS ‘Piñonet’ and BL) revealed similar results. Plants homozygous for the PI 414723 allele at the SNPCmND13bis and NDHRM26 markers were not all resistant. Some plants showed symptoms (albeit delayed) despite being homozygous for the allele of the resistant parent. Moreover, some of the plants homozygous for the allele of the susceptible parent in this region recovered from symptoms.

In a previous study performed by Siskos et al. [21], who sequenced a resistant *C*. *melo* line K18 and a susceptible *C*. *melo* line K15 (similar genetic background to K18 but lacking the ch11 QTL), four non-synonymous mutations were identified in MELO3C022319.2 of PI 414723.

Among the four mutations, two presented a correlation with ToLCNDV resistance: the melon accessions with T/C mutation at position 30,355,908 bp on chromosome 11 were consistently associated with symptomless phenotypes, while the C/T mutation on 30,354,702 bp chromosome 11 showed a correlation with intermediate resistance. Additionally, Siskos et al. [21] identified two more variants, T/G at 30,354,543 bp and C/A at 30,354,303 bp, that were not related to ToLCNDV resistance.

These four SNPs were not covered in our GBS results. Thus, the regions containing these four positions were sequenced for PI 414723, WM-7, PS ‘Piñonet’ and another susceptible ibericus variety named PS, ‘Pipa de Oro’, confirming the presence of the mutant alleles associated with resistance only in the resistant accessions (Table S6).

#### 2.2.4. QTLs Mapping and Expression Analysis Based on BSR-seq

1. Bulk construction and sequencing

Homozygous plants for the PI 414723 allele at the CPMSNP315, SNPCmND13bis and NDHRM26 SNP positions of the F_2_ (PI 414723 × PS ‘Piñonet’) and F_2_ (PI 414723 × BL) populations showed similar delayed appearance of mild symptoms, followed by an alternance of both recovery and the display of ToLCNDV symptomatology in young leaves. Since this behavior was absent in WM-7 after ToLCNDV infection, but the same major QTL in chromosome 11 was linked to this virus resistance in both accessions, we further searched for additional genomic regions involved in PI 414723 differential response to ToLCNDV.

We combined bulked-segregant analysis with whole transcriptome sequencing (BSR-seq) using the genotypic and phenotypic results obtained in this work. Five bulks of F_2_ (PI 414723 × PS ‘Piñonet’) plants were generated based on two criteria: (i) the genotype of the plants in chromosome 11 CMPSNP315 and CMPSNP475 (30,194,725 bp and 30,819,884 bp) in chromosome 11 SNP markers and (ii) symptoms and viral accumulation along the ToLCNDV disease course.

**Figure 4:**
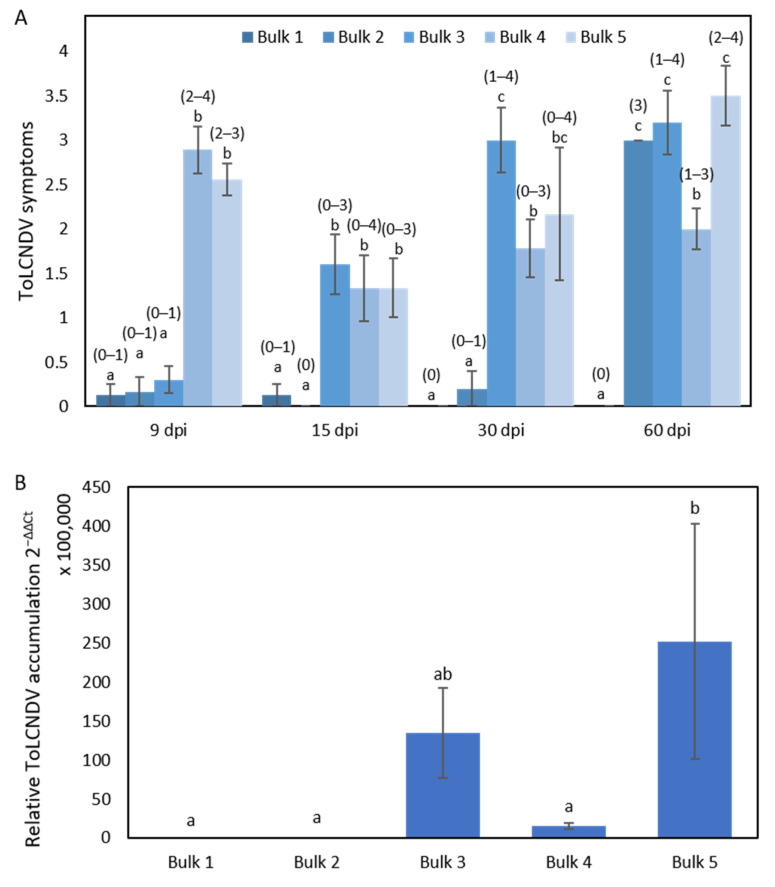
The responses in the five bulks from the F_2_ population derived from the cross PI 414723 × PS ‘Piñonet. (**A**) The average symptom score at 9, 15, 30 and 60 days post inoculation (dpi) with ToLCNDV in the five bulks of plants, with the range of symptoms shown above the bars, and (**B**) relative ToLCNDV accumulation in the five bulks (calculated as 2^−ΔΔCt^) at 15 dpi. Bars represent the standard error. Different letters indicate significant differences among genotypes at the same date (*p* < 0.05, LSD test).

Homozygous plants for the PI 414723 allele of chromosome 11 markers were classified into three bulks (Figure 4): (Bulk 1) plants fully resistant to ToLCNDV that remained asymptomatic and with low viral titers throughout all the assay (8 plants); (Bulk 2) plants that presented low viral titers but progressed from symptomless to achieve high symptoms at 60 dpi (6 plants); (Bulk 3) plants asymptomatic at the beginning of the assay that had high symptoms after 15 dpi that accumulated higher viral titers than bulks 1 and 2 (10 plants). Conversely, homozygous plants for the PS ‘Piñonet’ alleles, all with severe symptoms after 15 dpi, were grouped into Bulk 4 and Bulk 5 according to their low and high viral titers, respectively (9 and 6 plants, respectively) (Figure 4).

After Illumina RNA sequencing, a total of 577 million raw paired-end reads were generated for the five sequences (Table S7).

After the alignment of the sequences to melon reference genome, subsequent variant callings were performed to compare bulks representing the diverse responses of F_2_ (PI 414723 × PS ‘Piñonet’) plants to ToLCNDV infection. Ten datasets were generated, identifying an average of more than 132,000 SNPs in each of them, with more than 27,000 SNPs remaining after filtering by quality (Table S7). In all datasets, Δ(SNP-index) of the QTLseq method and the G′value were calculated and used to detect genomic regions associated with ToLCNDV response in PI 414723 (Figure S3, Table S8). To increase the reliability of the detections, only those QTLs identified by both statistical approaches were considered significant (*p* ≤ 0.05). According to this criterion, no QTLs were detected in comparison between Bulk 1 and Bulk 2, Bulk 2 and Bulk 3 and Bulk 4 and Bulk 5. However, across all other combinations, at least one QTL was identified (Table 4 and Table S8). Nine genomic regions were detected, being distributed over chromosomes 2, 5, 8, 9 and 11 of the PI 414723 melon accession associated with ToLCNDV resistance. We named the identified QTLs as a bulk comparison number combined with a chromosome number.

As was expected, a major QTL was identified on chromosome 11 when Bulk 1, Bulk 2 and Bulk 3 (plants with different resistance levels but homozygous for the resistant allele at the chromosome 11 CPMSNP315 marker) were compared with Bulk 4 or Bulk 5 (plants displaying severe ToLCNDV symptoms and PS ‘Piñonet’ homozygous at the CPMSNP315 position) (Figure S3, Table 4). Six QTLs were detected on this chromosome between 16,997,817 and 31,900,318 bp that overlapped with the interval of the QTL detected by CIM (29,176,476–30,819,944 bp). Among comparatives between pools, the highest G-prime and ∆SNP values were obtained for all the QTLs located on this chromosome, but 1 vs 3, in which the QTL located on chromosome 9 (covering a region from 633,982 to 13,389,235 bp), had the highest values (51.76 and −0.51 of the maximum values of G-prime and ∆SNP, respectively).

On chromosome 2, three independent regions were identified as linked to ToLCNDV resistance (Table 4). When we compared Bulk 3 with bulks 1 and 4, two overlapping QTLs were detected, covering a candidate region spanning from 3,580,909 to 6,945,099 bp. In contrast, after the comparison of Bulk 3 and Bulk 5, a nearby but not overlapping region was found, delimited between 9,669,827 and 10,218,866 bp. Additionally, the QTLs 2vs4.chr2, 2vs5.chr2 and 3vs5.chr2 overlapped between 21,065,243 and 21,339,968 bp in a region ranging from 12,066,407 to 26,957,490 bp that includes the region of chromosome 2 previously detected to be involved in resistance derived from WM-7 [16]. After those detected on chromosome 11, the QTLs 2vs4.chr2 and 2vs5.chr2 are the most relevant according to their G-prime and ∆SNP values.

Regions of two other chromosomes were identified. On chromosome 5, the QTL 2vs4.chr5 spanned from 23,851,469 to 25,788,776 bp. In chromosome 8, three QTLs were detected that were associated with Bulk 2 (2vs5.chr8; 2,826,416–3,834,866 bp) and Bulk 3 (3vs4.chr8.1 in the region 8,838,130–13,456,090 bp and 3vs4.chr8.2 in the region 14,028,052–23,893,540).

2. Differential expression analysis

To further investigate the resistance to ToLCNDV in the PI 414723 accession, we analyzed the whole transcriptome on the five bulks sequenced by RNA-seq. Focusing on the candidate region in chromosome 2 (Table 4), a differential expression analysis was performed by analyzing the expression patterns in PI 414723 and PS ‘Piñonet’. Nine genes were selected acording their location within or on overlapping regions of QTLs on chromosome 2, their previous association with plant response to viruses and their expression patterns across different bulks. The melon gene MELO3C015406.2, previously reported as up-regulated in the susceptible parental PS after ToLCNDV inoculation [19], was included in the analysis (Table 5).

**Figure 5:**
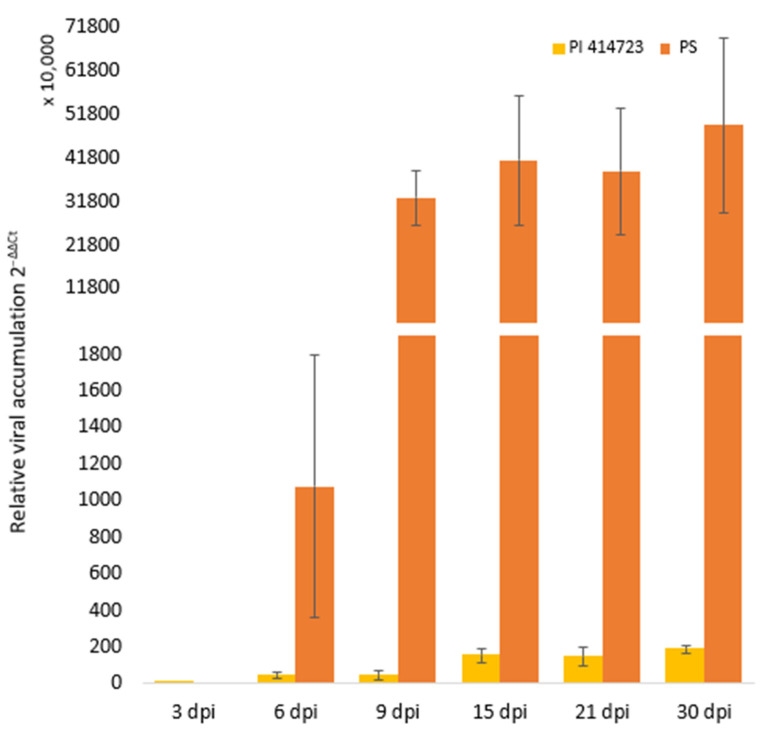
Temporal evolution of mean relative ToLCNDV accumulation in PI 414723 and PS ‘Piñonet’ (calculated as 2^−ΔΔCt^). Bars represent standard error.

To understand the molecular dynamics, we assessed the expression profiles of 10 candidate genes (Table 5) through qRT-PCR at seven different time points (0, 3, 6, 9, 15, 21 and 30 dpi), comparing their expression between the ToLCNDV-resistant source PI 414723 and the susceptible accession PS ‘Piñonet’. Most of PI 414723 plants remained asymptomatic or showed only moderate symptoms at 15 dpi. By the end of the trial, these plants exhibited a moderate virus accumulation at 30 dpi. Conversely, all PS ‘Piñonet’ plants were highly susceptible, displaying severe symptoms and a very high virus titer at 30 dpi (Figure 5).

**Figure 6:**
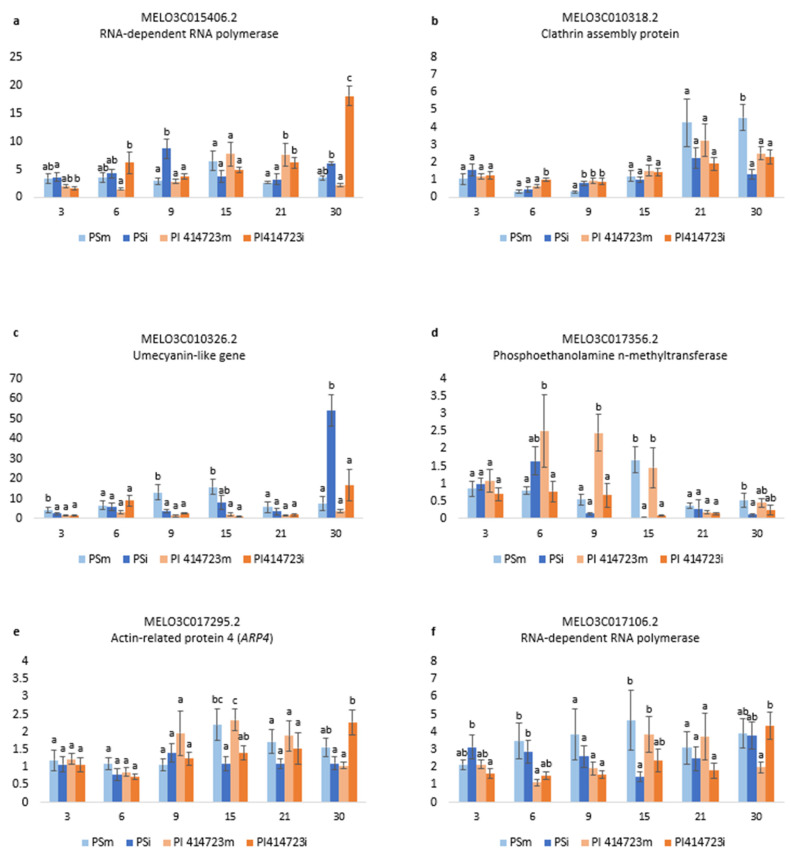
Relative expression 2^−ΔΔCt^ of (**a**) MELO3C015406.2, (**b**) MELO3C010318.2, (**c**) MELO3C010326.2, (**d**) MELO3C017356.2, (**e**) MELO3C017295.2 and (**f**) MELO3C017106.2 in plants inoculated with ToLCNDV (PSi and PI 414723i) and control plants (PSm and PI 414723m, mock-inoculated) at 3, 6, 9, 15, 21 and 30 dpi. Bars represent the standard error. Different letters in the same evaluation date indicate significant differences among genotypes (*p* < 0.05, LSD test).

Significant differences between susceptible and resistant accessions of ToLCNDV-inoculated and mock-inoculated plants were observed in the genes MELO3C015406.2, MELO3C010318.2, MELO3C010326.2, MELO3C017356.2, MELO3C017295.2 and MELO3C017106.2 (Figure 6). No relevant significant differences were found in the genes MELO3C017185.2, MELO3C017283.2, MELO3C017424.2 and MELO3C029682.2 (Figure S4).

Two RNA-dependent RNA polymerases were analyzed (MELO3C017106.2 and MELO3C015406.2). The expression pattern differed between both of them in the case of the susceptible genotype. MELO3C017106.2 was down-regulated in the ToLCNDV-inoculated plant of the susceptible PS with respect to the mock-inoculated plants at 15 dpi (Figure 6f). Conversely, the level of expression of MELO3C015406.2 in the virus-inoculated plants was higher than in the mock-inoculated plants at 9 dpi (Figure 6a). However, the level of expression of both MELO3C017106.2 and MELO3C015406.2 in the resistant PI 414723 virus-inoculated plants was higher than in the mock-inoculated of this genotype at 30 dpi (Figure 6a,f).

For clathrin assembly protein (MELO3C010318.2) the first differences in PS were observed at 9 dpi, when the level of expression in the PS ToLCNDV-inoculated plants was higher than in the mock-inoculated plants. However, the highest difference was observed at the later dates after inoculation. At 30 dpi, a reduction in the level of expression of this gene was observed in the virus-inoculated plants of the susceptible genotype PS with respect to the mock-inoculated plants, whereas an increase in gene expression was observed in ToLCNDV-inoculated plants of PI 414723 at 6 dpi (Figure 6b).

The pattern of expression of the umecyanin-like gene (MELO3C010326.2) changed with time in the susceptible PS, while no significant differences in the level of expression in both treatments were detected for PI 414723. At early dates after inoculation (3 dpi, 9 dpi and 15 dpi), this gene was down-regulated in the virus-inoculated plants of PS. However, at 30 dpi, there was an increase in the level of expression detected in ToLCNDV-inoculated plants of PS with respect to the mock-inoculated control (Figure 6c).

In the case of phosphoethanolamine n-methyltrasnferase (MELO3C017356.2), it was down-regulated in the virus-inoculated plants with respect to the mock-inoculated plants at 6 dpi, 9dpi and 15 dpi in the resistant PI 414723 and at 15 and 30 dpi in the susceptible PS (Figure 6d).

There were no differences in the expression of the actin-related protein 4 (*ARP4*) (MELO3C017295.2) in the first stages after infection between the different genotypes or between virus- and mock-inoculated plants. However, at 15 dpi, the level of expression in both PI 414723 and PS mock-inoculated plants increased, while it remained lower in virus-inoculated plants. At 30 dpi, the level of expression in the virus-inoculated PI 414723 was higher than in the mock-inoculated one (Figure 6e).

## 3. Discussion

### 3.1. WM-7

Several accessions belonging to different *C*. *melo* groups (acidulous, kachri, momordica and tibish) have been reported as ToLCNDV-resistant or -tolerant [13,18,29]. The WM-7 resistance source has been thoroughly studied. A major ToLCNDV resistance QTL, located on chromosome 11, was identified in segregant populations derived from this source [16]. The results obtained in this study agree with this previous research and allowed us to narrow down the major candidate region located on chromosome 11. This study also supports the idea of a major candidate gene for ToLCNDV resistance, the MELO3C022319.2, which encodes a DNA primase large subunit, previously suggested by Sáez et al. [19]. Our results provide candidate mutation modifiers of this gene, additional to those reported by Siskos et al. [21] and whose association with resistance needs to be further studied.

Previous studies showed the involvement of modifier regions controlling WM-7-derived resistance to ToLCNDV on chromosomes 2 and 12 when the locus on chromosome 11 was in heterozygosity. In a F_2_ population derived from the initial cross of WM-7 as a ToLCNDV-resistant source and a susceptible Piel de sapo, we observed that F_2_ plants heterozygous for the chromosome 11 marker D16 (position 30,246,542) exhibited lower viral titers when the CMPSNP658 marker on chromosome 2 was homozygous for the WM-7 allele compared to F_2_ plants homozygous for the PS allele at D16 [16].

Our F_3_ population is homozygous for the RC alleles in the candidate region on chromosome 12, which includes the marker AI_35-A08 (12,750,025). Sáez et al. [16] described an epistatic effect involving chromosome 12 and 11 regions, where F_2_ plants homozygous for PS at the chromosome 12 marker AI_35-A08 exhibited more severe symptoms when they were also homozygous for the PS allele at D16 compared to when they were heterozygous for the D16 marker on chromosome 11. We did not observe this behavior in our F_3_ population [16]. Here, we evaluated F_3_ plants derived from the cross between WM-7 and a traditional melon landrace of the Rochet market class. Plants were homozygous for the resistant parental allele for the markers of the candidate region of chromosome 2 and homozygous for the susceptible parental allele for the markers of the candidate region of chromosome 12. Although low levels of symptoms were observed in both homozygous for RC allele and heterozygous F_3_ (WM-7 × RC) plants, unexpectedly, viral titers were not reduced in plants presenting the locus of chromosome 11 in heterozygosity but the locus of chromosome 2 homozygous for the WM-7 allele. This discrepancy can be due to several factors, such as the different genetic background, and the occurrence of epistatic interaction between both 2 and 12 chromosomes loci determining ToLCNDV accumulation levels. Hence, the ongoing search for resistance genes within these minor QTLs is essential for a comprehensive understanding of the genetic complexities and factors influencing ToLCNDV resistance.

Despite the fact that a clear effect of the chromosome 2 and 12 candidate regions was not found in this population, the genotyping-by-sequencing of the F_3_ plants derived from the WM-7 × RC cross enabled the detection of three high-impact variants, polymorphic between the resistant and the susceptible parents in genes located in the candidate region of chromosome 2, notably two affecting the MELO3C017345.2 encode detoxification protein. The differential expression of detoxification protein during cucumber mosaic virus infection has been observed in yellow passion fruit (*Passiflora edulis*) [30]. The differential expression of the detoxification protein has also been observed in banana (*Musa acuminata)* during banana bunchy top virus infection [31], as well as in *Vigna mungo* during Mungbean yellow mosaic India virus (MYMIV) infection [32]. These observations across different plants suggest a potentially conserved mechanism of viral resistance involving detoxification protein pathways. Detoxification protein has been linked to the maintenance of ROS homeostasis in resistant plants [32]. Another variant was found that was affecting MELO3C017328.2 (stress up-regulated Nod 19 protein).

Additionally, five high-effect variants, polymorphic between WM-7 and RC, were found in the candidate region of chromosome 12 [16]. One of these variants is located in MELO3C004853.2, encoding 30S ribosomal protein S3. In *A. thaliana,* endogenous 30S ribosomal subunit protein S11 was reported to affect cucumber mosaic virus (CMV) viral replication [33]. In general, several ribosomal proteins interact with viral mRNA and proteins to participate in viral protein biosynthesis and regulate viral replication and infection in host cells. The majority of these interactions are essential for the translation and replication of the virus, promoting infection and accumulation of the virus, while a minority are involved in host cell defense signaling by activating the immune pathway against the virus [34]. The other four high-effect variants affect four genes that do not seem to have a role in resistance. None of the seven genes located in chromosomes 2 and 12 were previously found to be differentially expressed between WM-7 and PS after ToLCNDV infection [19].

### 3.2. PI 414723, Source of ToLCNDV Resistance

In addition to the analysis of WM-7, we studied the genetic control of the resistance to ToLCNDV identified in the *C. melo* Indian accession PI 414723. This accession was also identified as resistant after a screening of *C. melo* accessions against ToLCNDV [15], and the derived F_1_ hybrids were characterized [16]. Our results, obtained with additional ibericus backgrounds, are in accordance with those previously reported, with alternation of periods of recovery in PI 414723 and the derived F_1_ hybrids after crossing with two different susceptible cultivars (PS ‘Piñonet’ and BL). This behavior in PI 414723 has been reported after infection with other viral species. Indeed, plants derived from PI 414723 with resistance to watermelon mosaic virus (WMV) developed mosaic symptoms on inoculated leaves but eventually recovered from symptoms, and low or no virus was detected on younger leaves. Recovery was not clearly evident until around the fourth true-leaf stage, and it can be assessed by the appearance of symptoms and recovery in young plant tissue. [35]. Additionally, after zucchini yellow mosaic virus (ZYMV) inoculation, F_3_ plants derived from the initial cross (PI 414723 × ‘TopMark’) showed severe systemic infection on the first two or three leaves above the inoculation zone, as well as complete recovery [36]. This recovery can be the result of a different molecular resistance mechanism in PI 414723 compared with WM-7.

### 3.3. QTL Analysis

Two major overlapping QTLs were found in chromosome 11 for PI 414723-derived resistance to ToLCNDV, with one associated with symptom development at 9 dpi and a second one linked to viral accumulation at 30 dpi. Both QTLs overlapped with the candidate region for ToLCNDV resistance derived from WM-7 on this chromosome (30,159,412–30,377,414 bp). This region contains MELO3C022319.2 encoding the DNA primase large subunit, which is consistent with findings from Sáez et al. [19] and Siskos et al. [21], who reported MELO3C022319.2 as the most likely candidate resistance gene in WM-7 and K18 melon accessions, respectively. In fact, molecular markers associated with WM-7-derived resistance (SNPCmND13bis (30,347,864 bp) and NDHRM26 (30,358,926 bp)), designed close to the MELO3C022319.2 (30,347,186–30,355,934 bp), have proven to be efficient in MAS for the introgression of PI 414723-derived resistance in two different ibericus backgrounds, PS ‘Piñonet’ and BL, confirming the effect of the QTL across different genetic backgrounds.

In our study, the regions carrying each of the SNPs identified by Siskos et al. [21] in MELO3C022319.2 were detected after sequencing in the resistance sources PI 414723 and WM-7, as well as in the susceptible cultivars PS ‘Piñonet’ and PS ‘Pipa de Oro’. The SNPs identified between resistant and susceptible genotypes were identical to those reported by Siskos et al. [21], specifically T/C at the genomic position 30,355,908 bp, C/T (30,354,702 bp), T/G (30,354,543 bp) and C/A (30,354,303 bp), confirming the mutations reported by Siskos et al. [21] in two additional melon accessions. The first two changes were reported to be associated with the analyzed resistant melon accessions, leading to the loss of function of the DNA primase for the virus, while maintaining the function of the protein for the host plant [21].

### 3.4. Additional Regions of Interest

Inheritance of PI 414723-derived resistance was not explained with monogenic control. In fact, in segregating generations, not all plants homozygous for the alleles of PI 414723 for the associated markers were resistant. In order to explore other genetic regions in depth, BSR-seq was performed to identify QTLs associated with the resistance to ToLCNDV with five bulks selected for their response to ToLCNDV and for their genotype in the chromosome 11 candidate region (bulks 1, 2 and 3, homozygous for PI 414723 allele, and bulks 4 and 5, homozygous for PS ‘Piñonet’ allele). This study supports the occurrence of the major QTL of chromosome 11 in the same region as that of WM7-derived resistance (30,159,412–30,377,414 bp), as well as modifier regions of chromosome 2 (QTL 2vs4.chr2 (12,066,407–26,957,490 bp), 2vs5.chr2 (18,022,512–25,261,922 bp) and 3vs5.chr2 (21.065.243–21.339.968 bp)), also overlapping with those derived from WM-7 [16]. Our study indicates a major role of chromosome 2 regions compared with those of chromosome 12. In fact, additional regions of chromosome 2 were identified that had not been previously detected in WM-7 derived resistance (QTL 3vs4.chr2 (3,580,909–6,532,819 bp) and 3vs5.chr2.1 (9,669,827–10,218,866 bp)).

Interestingly, this study provided information about three additional regions not previously detected. One is found in chromosome 9 (QTL 1vs3.chr9 (633,982–13,389,235 bp)). The other two new regions are located in chromosome 5 (QTL 2vs4.chr5 (23.851.469–25.788.776 bp) and chromosome 8 ((QTL 2vs5.chr8 (2,826,416–3,834,866 bp), QTL3vs4.chr8.1 (8,838,130–13,456,090 bp) and QTL 3vs4.chr8.2 (14,028,052–23,893,540)). Some overlap with those previously reported in patents. QTL 2vs4.chr5 (23.851.469–25.788.776 bp) located on chromosome 5 overlap with two QTL patented in *C. melo* (Nunhems B.V. Patent: US20190225983A1; Semillas Fitó S. A. Patent: EP3238533A1). In addition, a patent is reported for a QTL on chromosome 9 (Semillas Fitó S. A. Patent: WO2017186920A1); however, this QTL (22,683,516–23,398,028 bp) does not overlap with the QTL 1vs3.chr9 (633,982–13,389,235 bp).

Focusing on the QTL 2vs4.chr2 of PI 414723, which overlaps with the candidate resistance region identified in WM-7 on chromosome 2 [16] (which seems to be one of the most impactful on the resistance), we studied the expression of nine genes placed at this location, and one, MELO3C015406.2, was reported as up-regulated in susceptible parental PS after ToLCNDV inoculation by Sáez el al. [19].

### 3.5. Differential Expression Analysis

Six genes were found to be differentially expressed. The overexpression of the RNA-dependent polymerase MELO3C015406.2 in the susceptible genotype at early stages after the infection found by Sáez et al. [19] was confirmed in our assay. However, we also found an induction of this gene in the resistant PI414723 genotype at later stages of infection that had not been previously reported. We found another RNA-dependent RNA polymerase, MELO3C017106.2, to be down-regulated in the susceptible PS-inoculated genotype at 15 dpi, as well as similarly up-regulated in the resistant genotype at the end of the assay.

Studies on *Nicotiana tabacum* and *A. thaliana* have demonstrated the critical role of RNA-dependent RNA polymerases (RDRs) in the antiviral defense mechanism [37,38]. Notably, loss-of-function mutations in *RDRs* have been linked to increased susceptibility to various plant viruses, higher viral RNA accumulation and reduced viral-derived siRNA levels in *Arabidopsis* [38]. Similarly, natural variants of host *RDRs* in *N. benthamiana* have been associated with heightened susceptibility to viral infections [39]. In *Cucumis sativus*, multiple *RDR1* genes are involved in virus resistance. Silencing *CsRDR1c* led to increased accumulation of ZYMV, indicating the involvement of *CsRDR1c* in defense [40]. MELO3C015406.2, identified as an RDR5 protein, shares sequence homology with the *Solanum chilense*-derived *Ty1/Ty3* gene [19]. The *Ty-1* and *Ty-3* alleles have been shown to exhibit similarities to *RDR3*, *RDR4* and *RDR5* in *Arabidopsis*, suggesting their potential involvement in double-stranded RNA (dsRNA) formation following tomato yellow leaf curl virus (TYLCV) inoculation. Interestingly, the resistance conferred by *Ty-1* and *Ty-3* is specific to geminiviruses. Additionally, the elevated expression levels of *Ty-1* at 30 dpi in resistant lines compared to susceptible lines after TYLCV inoculation agree with our observations [41]. Furthermore, *Salvia miltiorrhiza smRDR3* induction following CMV infection suggests its role in antiviral silencing [42].

Both RNA-dependent RNA polymerase MELO3C015406.2 and MELO3C017106.2 show a differential expression pattern that agrees with their role in ToLCNDV resistance, and the latter is located in the candidate region of chromosome 2 QTL 2vs4.chr2 (12,066,407–26,957,490 bp) in the PI 414723-derived population. Therefore, they are interesting candidates that should be further studied in this host–pathogen system.

The clathrin assembly protein, putative MELO3C010318.2, was up-regulated during the initial post-infection period in PI 414723 and down-regulated late after infection in the susceptible PS melon. This differential regulation could be related to the resistance response, as in another cucurbit, *C. moschata,* a clathrin assembly family protein (CmoCh09G004640), has been described as significantly associated with PRSV resistance [43] and clathrin-mediated endocytosis has been related to virus movement [44,45,46,47,48]. In the same way, we observed a significant increase in MELO3C010326.2 (an umecyanin-like protein) expression in the susceptible PS 30 days post ToLCNDV inoculation. Umecyanins are involved in redox reactions occurring during primary defense responses in plants [49]. Silencing umecyanin in *N. benthamiana* led to lower beet necrotic yellow vein virus (BNYVV) coat protein levels in leaves. Up-regulated miR398 expression may activate primary defense responses across plant species by targeting genes that include umecyanin (in *N. benthamiana*), influencing redox processes involved in defense response activation against viral infection [50].

The gene MELO3C017356.2 (phosphoethanolamine n-methyltransferase), already found to be deregulated in the susceptible genotype [19], was down-regulated in PI 414723 from 6 dpi, with this down-regulation being delayed to 15 dpi in PS. This protein, part of a stress-induced family [51], has homology with *A. thaliana* S-adenosyl-L-methionine-dependent methyltransferases [19]. Viral proteins like C2, C4 and HCPro interact with S-adenosyl-L-methionine-related enzymes, affecting methylation processes, influencing RNA-mediated gene silencing [52,53,54].

*ARP4* has been implicated in viral movement [55]. Román et al. [56] reported MELO3C017295.2, which encodes Actin-related protein 4 (*ARP4*), to be a key gene in ToLCNDV resistance, finding a higher expression in the susceptible PS compared with PI 414723. In our study, variations in the expression of MELO3C017295.2 between PI 414723 and PS samples were observed, although these differences were not statistically significant, due to variability among the technical replicates, until 30 dpi, when we observed higher expression in the PI 414723 ToLCNDV-inoculated sample.

The six genes identified in this study are of significant interest due to their previously reported association with resistance, their strategic location within candidate regions on chromosome 2, and their distinctive expression profiles. Future research will focus on narrowing down the candidate region on chromosome 2 and conducting functional analyses of these genes. This will enhance our understanding of the genetic basis of resistance and facilitate the introgression of ToLCNDV resistance.

## 4. Materials and Methods

### 4.1. Plant Material

#### 4.1.1. WM-7

With the objective of fine-mapping the region associated with the resistance to ToLCNDV from WM-7 accession (*C. melo*, kachri group) and designing useful markers for MAS, WM-7 was crossed with the traditional melon landrace ‘Rochet’, RC (BGV003718). The F_1_ generation was selfed to produce the F_2_ and F_3_ generations. Seven F_3_ plants previously phenotyped for resistance to ToLCNDV and both parents were genotyped by sequencing.

#### 4.1.2. PI 414723

The Indian accession PI 414723 (*C. melo*, momordica group), previously identified as resistant to ToLCNDV, was crossed with two different Spanish susceptible melon landraces (*C. melo*, ibericus group): ‘Piel de Sapo’, PS ‘Piñonet’ (BGV003729), and ‘Blanco’, BL (BGV000444). Seeds of PI 414723 were provided by USDA-NPGS, and seeds of the Spanish landraces were supplied by the COMAV genebank.

Populations available were as follows: 169 F_2_ plants and 22 BC _PS ‘Piñonet’_ plants derived from the PS ‘Piñonet’ × PI 414723 cross and 133 F_2_ plants and 98 BC_BL_ plants from the BL × PI 414723 cross. PI 414723 (resistant) and PS ‘Piñonet’ (susceptible) were also used to evaluate the differential expression after ToLCNDV inoculation.

### 4.2. Inoculation Method and Disease Assessment

#### 4.2.1. Inoculation Method

Virus inoculations were performed mechanically in plants at one true-leaf stage, as described in López et al. [15]. The virus used in the assays was obtained from symptomatic leaf extracts of a susceptible zucchini accession previously agroinfiltrated by injection with a ToLCNDV-ES-infectious clone, with a nucleotide identity of a 99% with the sequence of the Spanish isolates KF749224 and KF749225 [57]. Firstly, one cotyledon and the true leaf were dusted with carborundum 600 mesh. Thereafter, 1 g of infected zucchini leaf tissue was mashed in an iced mortar together with inoculation buffer [50 mM potassium phosphate (pH 8.0), 1% polyvinylpyrrolidone 10, 1% polyethylene glycol 6000, 10 mM 2-mercaptoethanol and 1% activated charcoal] in a 1:4 (*w*/*v*) proportion; the resultant homogenate was scrubbed with a cotton-bud stick. Plants were re-inoculated seven days after the initial inoculation, except for the plants that were included in the differential expression assay.

For the differential expression assay, 50 seedlings of PI 414723 and PS ‘Piñonet’ were mechanically inoculated with ToLCNDV-ES. The same number of plants were mock-inoculated following the same protocol but rubbing with only inoculation buffer and carborundum.

#### 4.2.2. Disease Assessment

1. Symptoms evaluation

Plants were grown in a climatic chamber until 30 days post inoculation (dpi). Symptoms of ToLCNDV were scored at 9, 15 and 30 dpi. Plants derived from the initial PI 414723 × BL cross received an additional symptoms score at 37 dpi in order to obtain a more accurate representation of the behavior of the resistant and susceptible plants, capturing any late-emerging symptoms or recovery phenomena that may not be evident at earlier stages. Selected plants derived from the initial PI 414723 × PS ‘Piñonet’ cross were transfered to a greenhouse and received an additional symptom score at 60 dpi. The visual scale used was the previously described [15], ranging from 0 (absence of symptoms) to 4 (very severe symptoms). According to this scale, plants scored below 2 were classified as resistant, while plants scored 2 or above were considered susceptible (Figure S5).

2. Viral accumulation analysis

Quantitative polymerase chain reaction (qPCR) was used to quantify the viral titer at 15 dpi and/or 30 dpi in the populations (Results, Section 2.1.1, Section 2.1.2, Section 2.2.1 and Section 2.2.3) and at 3, 6, 9, 15, 21 and 30 dpi in the plants used to study the gene expression patterns (Result, Section 2.2.4. Three technical replicates per plant and date were analyzed. Amplifications were carried out with primers designed from the DNA-A of isolates KF749223, ToLCNDVF1 (5′-AATGCCGACTACACCAAGCA-3′, positions 1145–1164 bp) and ToLCNDVR1 (5′-GGATCGAGAAGAGAGTGGCG-3′, positions 1399–1418 bp), producing a fragment of 274 bp. The single-copy melon gene *CmWIP1* (CmWIP1F; 5′-TAGGGCTTCCAACTCCTTCCTCTT-3′ and CmWIP1R: 5′CTTGCAATTGATGGGTGTGATCTTCTTG-3′) was used as an internal control [16]. The qPCR was performed in a Roche LightCycler 480 apparatus. The reaction mix contained 7.5 μL of 2X iTaqTM universal SYBR^®^ Green Supermix (Bio-Rad Laboratories Inc., Waltham, MA, USA), 1.5 μL of each primer (10 mM), 1.5 μL of water and 3 μL of total DNA (15 ng/µL). Cycling conditions consisted of incubation at 95 °C for 5 min and 40 cycles of 95 °C for 5 s and 60 °C for 30 s. Relative accumulation of ToLCNDV was estimated by the 2^−ΔΔCt^ method [58].

3. Tissue printing

For the additional detection of ToLCNDV in populations derived from crosses between PI 414723 and PS ‘Piñonet’ (Results, Section 2.2.2), a petiole was detached from each plant at 30 dpi, and the cross-sections were blotted onto positively charged nylon membranes (Hybond-N, Amersham Biosciences, Freiburg, Germany). Subsequently, the membranes were air-dried, covalently fixed by UV cross-linking (700 × 100 mJ/cm^2^) and hybridized by a digoxigenin-labeled ToLCNDV-RNA probe. The pre-hybridization, hybridization and washing of the membranes were conducted as described previously [59].

4. DNA extraction

Total DNA was extracted from the youngest leaves showing symptoms at 15 dpi and 30 dpi following the cetyltrimethyl ammonium bromide (CTAB) method [60]. DNA was quantified using spectrophotometry in a NanoDrop ND-1000 spectrophotometer v.3.5 (Thermo Fisher Scientific, Waltham, MA, USA). Subsequently, DNA was diluted with sterile purified water to a final concentration of 50 ng/µL for the HRM analysis and 5 ng/µL for the qPCR analysis.

### 4.3. Molecular Markers Analysis

#### 4.3.1. WM-7

To narrow down the candidate region, seven F_3_ (WM-7 × RC) plants were genotyped by sequencing (GBS) after being evaluated for ToLCNDV resistance.

GBS libraries were prepared as previously described [61], using the restriction enzyme MsII on the Illumina NovaSeq 6000 SP FC platform (Illumina Inc., San Diego, CA, USA) in LGC Genomics GmbH (Berlin, Germany). Raw reads were then quality-filtered, adapted, enzyme-clipped and processed (2 × 150 bp). The high-quality paired-end reads were mapped to the latest version of melon reference genome (v4.0) using Bowtie2 v2.3.4.1 [62]. SAM files were converted to BAM format with Samtools v1.11 [63], SNP calling was performed using Freebayes v1.3.4 [64] and Vcftools v0.1.16 [65] was used to filtrate the raw SNPs with alternate count (--min-alternate-count 3), mapping quality (--min-mapping-quality 10), base quality (--min-base-quality 20) and coverage (--min-coverage 3). The SNPs identified with the GBS in the candidate region on chromosome 11 were used to design new high-resolution melting (HRM) markers (Table S3).

To perform HRM genotyping, DNA was amplified by PCR in a total volume of 10 μL containing 2 μL of mix (HOT FIREpol EvaGREEN HRM Mix (NO rox.5X)) (Solis BioDyne, Tartu, Estonia), with 0.3 μL of each primer (10 mM), 5.4 μL of water and 2 μL of total DNA (50 ng/µL). Amplification was carried out in a Roche LightCycler 480 thermocycler (Roche, Basel, Switzerland) with the following program: initial denaturation at 95 °C for 12 min, followed by 45 cycles of denaturation at 95 °C for 15 s, annealing of 20 s at 60 °C and extension at 72 °C for 20 s.

The HRM markers previously mentioned were used to verify the linkage to ToLCNDV resistance in the segregating F_3_ population from the cross WM-7 × RC.

#### 4.3.2. PI 414723

The different segregating populations were genotyped using previously available and new SNP panels.

Initially, an existing panel of 124 SNP markers evenly distributed across the genome was implemented on the Agena Bioscience iPLEX^®^ Gold MassARRAY platform by the Epigenetic and Genotyping Unit of the University of Valencia (Unitat Central d’Investigació en Medicina (UCIM), Valencia, Spain) and used to genotype the F_2_ (PI 414723 × PS ‘Piñonet’) population. This SNP set had been previously validated in populations derived from occidental melons (ibericus or cantalupensis) × oriental melons (momordica, makuwa, acidulus, kachri, etc.) crosses [16,24,25,26,27,28].

The HRM markers designed from the GBS (described in Section 4.3.1) were used to verify the linkage to ToLCNDV resistance in the segregating populations developed from the resistance source PI 414723, F_2_ and BC populations from the crosses PI 414723 × PS ‘Piñonet’ and PI 414723 × BL.

### 4.4. Data Analysis

The chi-square (*χ^2^*) test was used to assess whether the number of resistant/susceptible plants fitted the expected segregation. Analyses of variance (ANOVA) were performed. Comparisons of means were made using the LSD test with a probability level of *p* < 0.05. Statistical analyses were conducted using Statgraphics Centurion XVI.I software v19 (StatPoint Technologies, Inc., Warrenton, VA, USA).

### 4.5. QTL Analysis

Linkage analysis and map construction were performed with JoinMap v4.0 [66] using the Kosambi mapping function.

A Kruskal–Wallis test [67] was performed using the genotype data of 78 SNP markers and the phenotypic data of the F_2_ (PI 414723 × PS ‘Piñonet’) population using MapQTL v6.0 software. A threshold of *p* < 0.05 was set to identify markers significantly associated with ToLCNDV resistance. Symptom scores at 9, 15 and 30 dpi and virus titer at 30 dpi were used to perform composite interval mapping (CIM) using QGene software v4.4.0 [68].

### 4.6. F_2_ PI 414723 × PS ‘Piñonet’ RNA-BSR-seq Analysis

An RNA-BSR-seq analysis was conducted using five bulks constructed according to their genotype at CPMSNP315 and CPMSNP475 markers on chromosome 11, previously associated with ToLCNDV resistance and symptoms and viral accumulation along the ToLCNDV disease course.

Samples were prepared by bulking the leaf tissue at 9 dpi. The 5 samples of processed leaf tissue (F_2_ PI 414723 × PS) were sent to Genomics4All (Universidad Politécnica de Madrid, Madrid, Spain) for cDNA library construction. RNA extraction, DNAse treatment, RNA purification, quality checking, the construction of RNA-seq libraries and cDNA synthesis were performed following the procedure previously described by Sáez et al. [19]. RNA-seq libraries were sequenced (single-end 50 pb) on a HiSeq 2500 Illumina (Illumina, San Diego, CA, USA).

#### 4.6.1. BSR-seq Data Analysis

Raw-read quality was checked by FastQC v0.12.0 [69] and aligned to the last version of melon reference genome (v. 4.0) available on the melonomics.net website [70] using HISAT2 software v2.2.0 [71]. SAMtools v1.11 [72] was used to convert SAM files to BAM files and sort them. SNPs were called with Freebayes v1.3.4 [64], filtering by base quality, mapping quality and base count, generating a VCF file for each comparative between bulks. Filtering by minor allele frequency (MAF) and maximum missing count was carried out with VCFtools v0.1.16 [65] to avoid low-confident SNPs. All the software and commands were run in a Linux server environment.

BSA was performed with QTLseqr v0.7.5.2, an R v4.3.2 package for bulked-segregant analysis. Ten different comparative analyses of the 5 bulks were performed (1 and 2; 1 and 3; 1 and 4; 1 and 5; 2 and 3; 2 and 4; 2 and 5; 3 and 4; 3 and 5; 4 and 5) using SNP calling data. Filtering was then performed according to recommendations [73].

REF_FREQ, SNP index and read depth at the position (DP) were determined before and after the filtering to assess filtering thresholds and check the quality of our data. Finally, both QTL-seq analysis and G-prime analysis were performed to identify potential QTLs. A threshold of *p* < 0.05 was set to identify QTLs significantly associated with ToLCNDV resistance. Only QTLs detected by both methods were selected. The tricube-weighted ∆(SNP-index), G-prime values and −log10(*p*-value) (derived from G-prime value) were plotted.

#### 4.6.2. Transcript-Level Expression Analysis

Genomics4all carried out the procedure described by Pertea et al. [74] in order to obtain the fragments per kilobase of exon per million mapped fragments (FPKM) for the different bulks. Read quality of the raw reads was checked using FastQC v0.12.0 [69]. HISAT2 software v2.2.0 [71] was used to align clean reads to the latest version of the melon reference genome available at the time of analysis (v. 3.6.1), which is located on the melonomics.net website [70]. SAMtools v1.7 [72] was used to convert SAM files to BAM files and sort them. Expression values were obtained using StringTie2 v2.1.1 [75]. Ballgown v3.6.3 [76] was used to organize, visualize and analyze the expression measurements. Transcripts with 0 values in all the bulks were eliminated. Log2 (fold changes) were calculated for all the bulks combinations; log2 (fold changes) > 1 and <−1 were considered significant.

#### 4.6.3. Expression Differences Analysis

Nine genes were selected according to different criteria (Table 5): their expression patterns across different bulks, their location within or on overlapping regions of QTLs located on chromosome 2 and their previous association with plant response to viruses. The melon gene MELO3C015406.2, previously reported as up-regulated in the susceptible parental PS after ToLCNDV inoculation [19], was included in the analysis.

The expression level of ten candidate genes potentially associated with ToLCNDV resistance in melon was evaluated via qRT-PCR at seven different time points (0, 3, 6, 9, 15, 21 and 30 dpi) At each time point, 12 plants were assessed, consisting of 6 plants per genotype; within each genotype, 3 plants were ToLCNDV-inoculated and 3 plants were mock-inoculated.

The expression patterns of these ten genes were confirmed using three biological replicates of both mock- and ToLCNDV-inoculated plants of PI 414723 and PS ‘Piñonet’.

Specific primer pairs were designed using sequences from the melon_v4.0 melo genome databases available on the Melonomics Database [70]. Primer design, accounting for homologous sequences, was conducted using the online software Primer3web (version 4.1.0) [77]. Each set of primers designed for the candidate genes successfully amplified 150 base-pair-length fragments at an optimal melting temperature (Tm) of 60 °C with GC contents ranging between 35% and 65%.

RNA was extracted with the use of TRITidy G Reagent (AppliChem, Darmstadt, Germany) according to the manufacturer’s instructions. Quality checking, DNAse treatment and cDNA synthesis was performed following the procedure previously described in Sáez et al. [19] qPCR experiments were carried out on a Roche LightCycler 480 instrument (Roche, Basel, Switzerland). The reaction mixture consisted of 7.5 μL of 2X iTaqTM universal SYBR^®^ Green Supermix (Bio-Rad), 1.5 μL of each primer (10 mM) and 1.5 μL of the cDNA sample in a total volume of 15 μL. Cycling conditions included an initial incubation at 95 °C for 5 min, followed by 40 cycles of 95 °C for 5 s and 60 °C for 30 s. Three technical replicates were performed for each sample.

Expression levels were analyzed using the relative quantitative accumulation by the 2^−ΔΔCt^ method [58]. As endogenous control, the melon Peptidyl-prolyl cis-trans isomerase gene (Cyclophilin CYP7, MELO3C025848.2) [78] was utilized as a reference for the expression level of each candidate gene under all conditions. Samples at 0 dpi were used as a calibrating sample.

### 4.7. DNA Primase Large Subunit Sequencing

Primers for sequencing the four reported MELO3C022319.2 changes, identified by Siskos et al. [21], were designed using MELO3C022319.2 sequence from the melon_v4.0 melo genome databases available at on the Melonomics Database [70] and the online software Primer3web (version 4.1.0) [77]. DNA was extracted from resistance (PI 414723, WM-7) and susceptible (PS ‘Piñonet’, PS ‘Pipa de Oro’) genotypes, following the mentioned protocols [60]. The designed primers were PCR-validated for specificity and subsequently used for sequencing at the DNA Sequencing Service (DNA Sequencing Service, Instituto de Biología Molecular y Celular de Plantas, IBMCP-UPV, Valencia, Spain).

## 5. Conclusions

The findings here obtained validate the locus on chromosome 11 linked to ToLCNDV resistance, showing the DNA primase large subunit coding gene as the main candidate gene for resistance, with non-synonymous mutations detected in PI 414723 and WM-7. Despite the focus on chromosome 11, the identification of other relevant regions provides additional insights into the resistance mechanism. Furthermore, the analysis of gene expression patterns on chromosome 2 reveals potential susceptibility-related candidates.

## Figures and Tables

**Table 1:** The genotypes for the SNPs in the candidate regions controlling the ToLCNDV resistance of chromosomes 2, 11 and 12 of the F_2_ (WM-7 × RC) plant selected to be selfed (A: homozygous for RC allele; H: heterozygous; B: homozygous for WM-7 allele).

Markers	Position (bp) ^1^	Chr ^2^	F_2_-WM-7 × RC
SNPCmND1	23,984,243	2	B
SNPCmND2	25,291,938	2	B
SNPCmND3	25,448,714	2	B
SNPCmND4	25,611,353	2	B
SNPCmND5bis	25,904,727	2	B
SNPCmND6	26,504,936	2	B
SNPCmND7	30,249,798	11	H
SNPCmND9	30,276,354	11	H
SNPCmND11	30,280,636	11	H
SNPCmND13bis	30,347,863	11	H
SNPCmND15	30,377,414	11	A
SNPCmND14	30,395,841	11	A
SNPCmND16bis	30,403,862	11	A
SNPCmND17	30,410,536	11	A
SNPCmND19	30,441,821	11	A
SNPCmND20	30,458,337	11	A
SNPCmND22	30,472,365	11	A
SNPCmND23	30,482,001	11	A
SNPCmND25	30,537,322	11	A
SNPCmND26bis	10,175,361	12	A
SNPCmND27	11,965,753	12	A
SNPCmND28bis	13,551,907	12	A
SNPCmND29	14,425,696	12	A
SNPCmND30	15,368,098	12	A

^1^ Physical position in the last version of the melon genome v4.0, available at https://www.melonomics.net/ (accessed on 21 November 2023); ^2^ Chromosome.

**Table 2:** Genotype for the candidate region on chromosome 11 according to the genotyping-by-sequencing of F_3_ plants derived from the initial cross WM-7 × RC, selected after ToLCNDV resistance evaluation (A: homozygous for RC allele; H: heterozygous; B: homozygous for WM-7 allele). The phenotype is indicated (S: susceptible; R: resistant).

Marker	Position (bp)	8	4	11	17	14	7	4b
S11_30159412	30,159,412	B	B	H	A	B	H	H
S11_30191060	30,191,060	B	B	H	A	H	H	H
S11_30191187	30,191,187	B	B	H	A	H	H	H
S11_30191253	30,191,253	B	B	H	A	H	H	H
S11_30191271	30,191,271	B	B	H	A	H	H	H
S11_30191293	30,191,293	B	B	H	A	H	H	H
S11_30197124	30,197,124	B	B	H	A	H	H	H
S11_30197480	30,197,480	B	B	H	A	A	H	H
S11_30197584	30,197,584	B	B	H	A	H	H	H
S11_30197679	30,197,679	B	B	H	A	H	H	H
S11_30197706	30,197,706	B	B	H	A	H	H	H
S11_30197725	30,197,725	B	B	H	A	H	H	H
S11_30197739	30,197,739	B	B	H	A	H	H	H
S11_30202708	30,202,708	B	B	H	A	H	H	H
S11_30202743	30,202,743	B	B	H	A	H	H	H
S11_30202810	30,202,810	B	B	H	A	H	H	H
S11_30202831	30,202,831	B	B	H	A	H	H	H
S11_30216956	30,216,956	B	B	H	A	H	H	H
S11_30217175	30,217,175	B	B	H	A	H	H	H
S11_30221819	30,221,819	B	B	H	A	H	H	H
S11_30221970	30,221,970	B	B	H	A	H	H	H
S11_30227392	30,227,392	B	B	H	A	H	H	H
S11_30261314	30,261,314	B	B	H	A	H	H	H
S11_30287824	30,287,824	B	B	H	A	H	A	H
S11_30287838	30,287,838	B	B	H	A	H	A	H
S11_30287875	30,287,875	B	B	H	A	H	A	H
S11_30307415	30,307,415	B	B	H	A	H	H	H
S11_30307457	30,307,457	B	B	H	A	H	H	H
S11_30339408	30,339,408	B	B	H	A	H	H	H
S11_30358926	30,358,926	B	B	H	A	H	H	H
S11_30359039	30,359,039	B	B	H	A	H	H	H
S11_30401768	30,401,768	A	A	A	A	A	A	A
Phenotype		R	R	S	S	S	S	S

**Table 3:** Quantitative trait loci (QTLs) identified in the F_2_-segregating population derived from the cross between PI 414723 and PS ‘Piñonet’, genotyped with SNPs distributed throughout the genome and phenotyped for resistance to ToLCNDV.

				Composite Interval Mapping					Kruskal-Wallis	
Trait ^1^	Chr ^2^	Interval 1.5 (cM) ^3^	Nearest Marker ^4^	LOD ^5^	Add ^6^	Dom ^7^	d/a ^8^	R ^9^	Mean PI 414723 ^10^	Mean PS ^11^
9 DPI	11	103.8–131.2 29,176,476–30,819,884 bp	CMPSNP315	8	0.799	0.651	0.815	20.2	0.545	1.917
30 DPI VT	11	103.8–131.2 29,176,476–30,819,884 bp	CMPSNP315	4.98	0.612	0.409	1.252	13.7	1.256	2.392

^1^ Symptom evaluation (9 dpi) and tissue printing virus titer (30 dpi VT) for the F_2_-segregating population. ^2^ Chromosome. ^3^ Interval position of the putative QTL, identified by composite interval mapping (CIM) in cM and bp on the genetic and physical maps according to a LOD drop of 1.5. The physical position (v4.0) is defined by the positions of the markers flanking the QTL interval. ^4^ Closest marker to the LOD peak. ^5^ Higher logarithm of the odds score. ^6^ Additive effect. ^7^ Dominant effect. ^8^ Add/ Dom. ^9^ R^2^ percentage of phenotypic variance explained by the QTL. ^10^ Mean of the genetic class PI 414723 for the corresponding marker. ^11^ Mean of the genetic class PS ‘Piñonet’ for the corresponding marker.

**Table 4:** QTLs detected linked to ToLCNDV resistance in bulks obtained from the F_2_ population derived from the cross PI 414723 × PS ‘Piñonet’.

			G-Prime Method Analysis				QTLseq Method Analysis			
Bulks	QTL Name	Chr ^1^	Start ^2^	End ^3^	Max G-Prime ^4^	Pos	Start ^6^	End ^7^	Peak	Pos Peak
Comparation						MaxG-Prime ^5^			∆SNP ^8^	∆SNP ^9^
1 vs. 3	1vs3.chr2	chr02	3,877,068	6,945,099	33.58	5,228,546	4,405,764	5,962,440	−0.42	5,355,437
	1vs3.chr9	chr09	633,982	13,389,235	51.76	6,725,288	2,480,077	9,259,367	−0.51	6,752,980
							10,249,392	10,603,982	−0.39	10,506,551
1 vs. 4	1vs4.chr11	chr11	29,046,233	31,900,318	112.82	31,900,318	30,571,554	31,399,076	0.59	30,904,594
1 vs. 5	1vs5.chr11	chr11	27,931,306	31,900,318	132.74	29,907,303	30,752,546	31,146,927	0.58	30,904,594
2 vs. 4	2vs4.chr2	chr02	12,066,407	26,957,490	100.79	23,226,160	12,635,695	15,275,207	0.48	14,509,192
							18,855,678	22,821,114	0.54	21,096,983
							24,325,575	25,333,267	0.53	24,858,784
	2vs4.chr5	chr05	23,851,469	25,788,776	41.77	24,970,077	24,710,571	25,611,014	0.43	24,970,077
	2vs4.chr11	chr11	25,706,075	31,900,318	158.85	30,403,683	30,571,554	31,417,092	0.58	30,904,594
2 vs. 5	2vs5.chr2	chr02	18,022,512	25,261,922	98.31	21,866,865	16,291,849	22,874,524	0.67	21,150,416
							24,345,760	25,203,049	0.47	24,858,784
	2vs5.chr8	chr08	3,341,340	3,834,866	46.44	3,655,973	2,826,416	3,746,350	0.43	3,132,745
	2vs5.chr11	chr11	26,303,416	31,900,318	144.67	30,404,623	30,442,913	31,415,732	0.60	30,904,594
3 vs. 4	3vs4.chr2	chr02	3,580,909	6,532,819	25.49	4,547,718	4,442,145	4,444,641	0.38	4,442,145
	3vs4.chr8.1	chr08	8,323,310	13,456,090	39.19	11,123,496	8,838,130	12,710,777	−0.44	10,503,405
	3vs4.chr8.2	chr08	14,028,052	23,893,540	41	18,282,774	18,282,774	19,070,463	−0.43	18,734,123
	3vs4.chr11	chr11	16,997,817	31,804,037	109.9	29,814,321	30,571,554	31,500,362	0.55	30,814,948
3 vs. 5	3vs5.chr2.1	chr02	9,669,827	10,218,866	33.58	9,936,717	9,936,717	10,062,220	0.37	9,936,717
	3vs5.chr2.2	chr02	21,065,243	21,339,968	32.26	21,122,957	20,979,784	21,339,968	0.39	21,277,282
	3vs5.chr11	chr11	24,679,309	31,804,037	150.28	29,324,169	30,571,554	31,293,094	0.51	30,818,944

Chr ^1^—chromosome. Start ^2^—position of the first SNP that passed the false discovery rate (FDR) threshold in G-prime method analysis. End ^3^—position of the first SNP that passed the FDR threshold in G-prime method analysis. Max G-prime ^4^—the max G’ score in the region. Pos MaxG-prime ^5^—the genomic position of the maximum G’ value in the QTL. Start ^6^—the position of the first SNP that passed the threshold in QTLseq method analysis. End ^7^—the position of the first SNP that passed the threshold in QTLseq method analysis. Peak ∆SNP ^8^—the ∆(SNP-index) value at the peak summit (QTLseq method analysis). Pos Peak ∆SNP ^9^—the position of the maximum value ∆(SNP-index) in the QTL (QTLseq method analysis).

**Table 5:** Candidate genes selected for expression analysis using qRT-PCR.

Gene Name	Position (bp)	Description	QTL
MELO3C015406.2	1,367,327–1,381,672	RNA-dependent RNA polymerase	-
MELO3C010318.2	17,006,652–17,008,337	Clathrin assembly protein. Putative	2vs4.chr2
MELO3C010326.2	17,122,601–17,123,944	umecyanin-like	2vs4.chr2
MELO3C029682.2	18,969,012–18,975,891	Transcriptional corepressor LEUNIG	overlapping region between QTLs 2vs4.chr2 and 2vs5.chr2
MELO3C017424.2	23,325,650–23,329,684	Transcription factor *bHLH35*	overlapping region between QTLs 2vs4.chr2 and 2vs5.chr2
MELO3C017356.2	23,965,259–23,968,861	Phosphoethanolamine n-methyltransferase	overlapping region between QTLs 2vs4.chr2 and 2vs5.chr2
MELO3C017295.2	24,499,946–24,508,159	Actin-related protein 4	overlapping region between QTLs 2vs3.chr2, 2vs4.chr2, 2vs5.chr2 and 3vs4.5
MELO3C017283.2	24,603,206–24,603,619	Transmembrane protein, putative	overlapping region between QTLs 2vs4.chr2 and 2vs5.chr2
MELO3C017106.2	25,821,307–25,826,094	RNA-dependent RNA polymerase	2vs4.chr2
MELO3C017185.2	25,323,802–25,325,467	NAC domain protein	2vs4.chr2

## Data Availability

All data are available in the manuscript and the Supplementary Materials.

## Supplementary Materials

The following supporting information can be downloaded at: https://www.mdpi.com/article/10.3390/ijms25168880/s1.
