# Supplementary material for: Genetic Dissection of ToLCNDV Resistance in Resistant Sources of Cucumis melo
Source: Int J Mol Sci. 2024 Aug 15;25(16):8880. doi: 10.3390/ijms25168880 (PMC11354858; doi:10.3390/ijms25168880)
Supplement: Supplementary file 1 [file ijms-25-08880-s001.zip › Figure S1.pptx]

## Slide 1
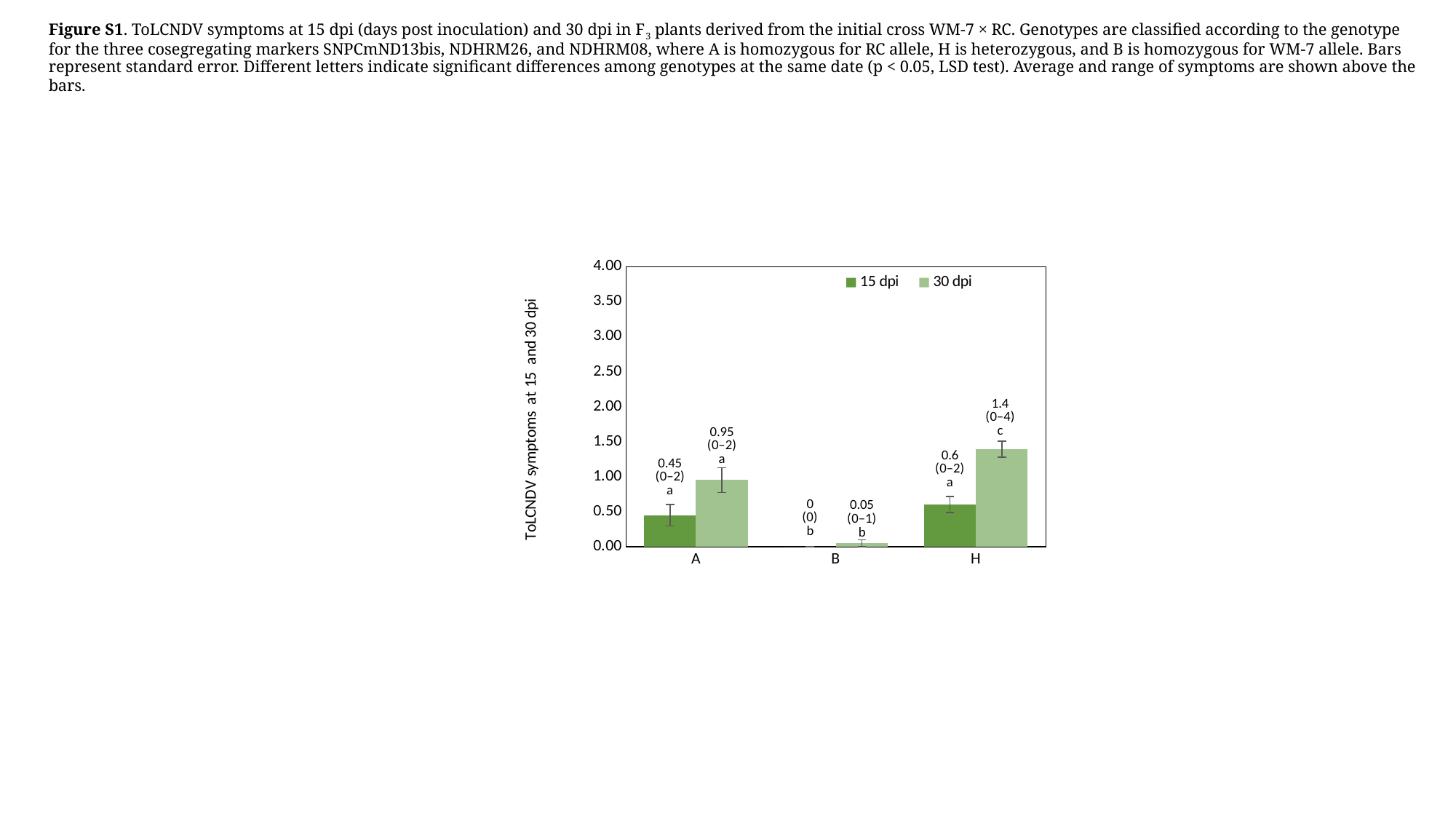

Figure S1. ToLCNDV symptoms at 15 dpi (days post inoculation) and 30 dpi in F3 plants derived from the initial cross WM-7 × RC. Genotypes are classified according to the genotype for the three cosegregating markers SNPCmND13bis, NDHRM26, and NDHRM08, where A is homozygous for RC allele, H is heterozygous, and B is homozygous for WM-7 allele. Bars represent standard error. Different letters indicate significant differences among genotypes at the same date (p < 0.05, LSD test). Average and range of symptoms are shown above the bars.
### Chart
| Category | 15 dpi | 30 dpi |
|---|---|---|
| A | 0.4523809523809524 | 0.9523809523809523 |
| B | 0.0 | 0.05 |
| H | 0.6046511627906976 | 1.3953488372093024 |
