# Supplementary material for: Genetic Dissection of ToLCNDV Resistance in Resistant Sources of Cucumis melo
Source: Int J Mol Sci. 2024 Aug 15;25(16):8880. doi: 10.3390/ijms25168880 (PMC11354858; doi:10.3390/ijms25168880)
Supplement: Supplementary file 1 [file ijms-25-08880-s001.zip › Figure S2.pptx]

## Slide 1
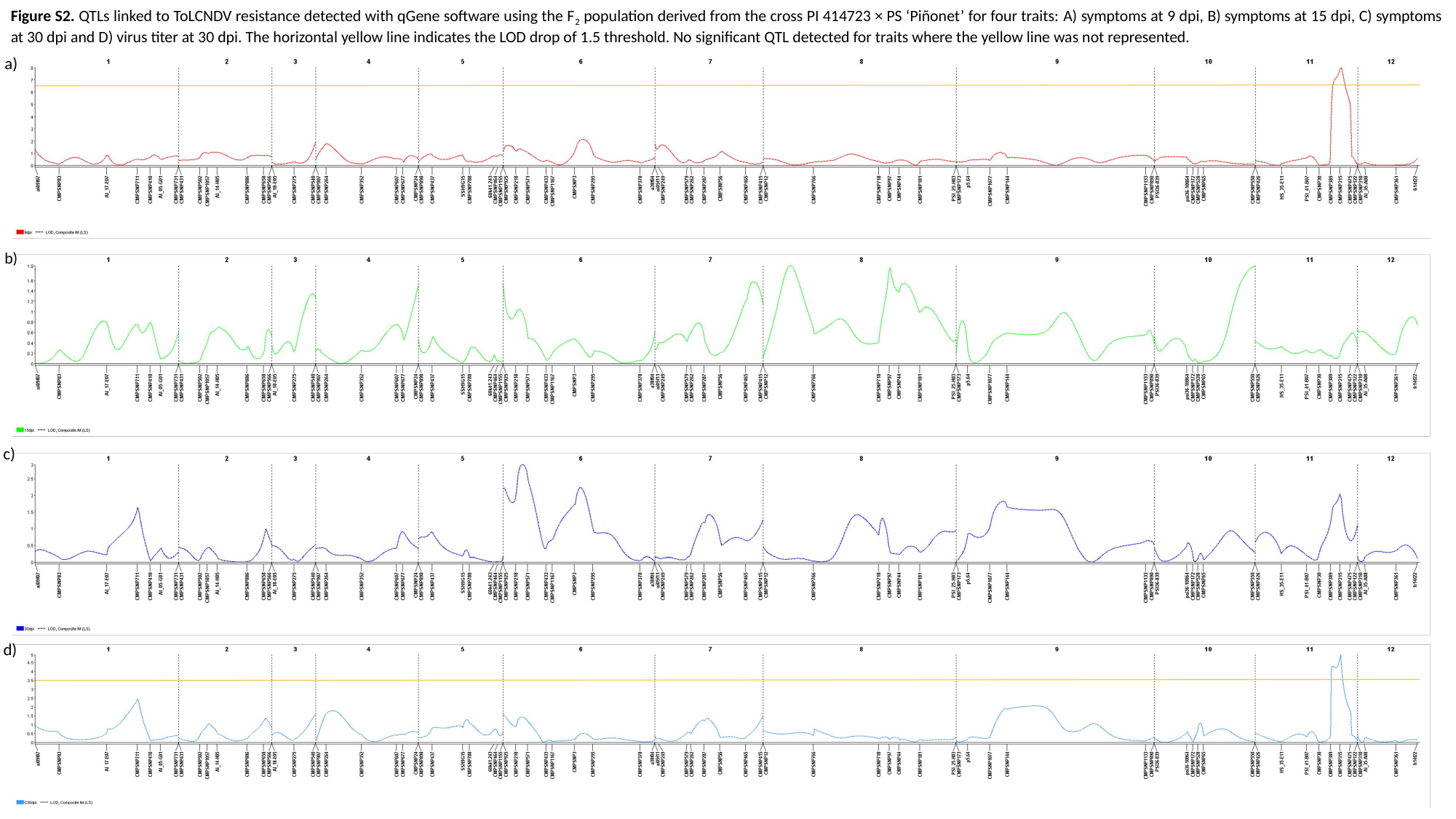

Figure S2. QTLs linked to ToLCNDV resistance detected with qGene software using the F2 population derived from the cross PI 414723 × PS ‘Piñonet’ for four traits: A) symptoms at 9 dpi, B) symptoms at 15 dpi, C) symptoms at 30 dpi and D) virus titer at 30 dpi. The horizontal yellow line indicates the LOD drop of 1.5 threshold. No significant QTL detected for traits where the yellow line was not represented.
a)
b)
c)
d)
