# Supplementary material for: Genetic Dissection of ToLCNDV Resistance in Resistant Sources of Cucumis melo
Source: Int J Mol Sci. 2024 Aug 15;25(16):8880. doi: 10.3390/ijms25168880 (PMC11354858; doi:10.3390/ijms25168880)
Supplement: Supplementary file 1 [file ijms-25-08880-s001.zip › Figure S3.pptx]

## Slide 1
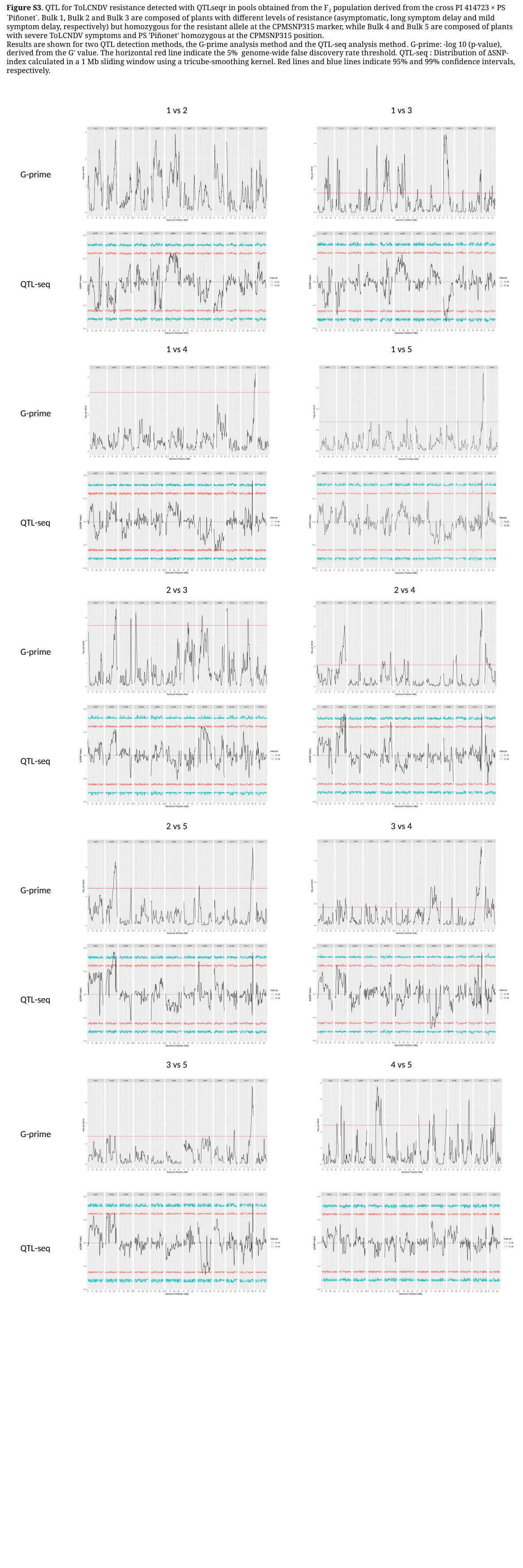

Figure S3. QTL for ToLCNDV resistance detected with QTLseqr in pools obtained from the F2 population derived from the cross PI 414723 × PS ´Piñonet´. Bulk 1, Bulk 2 and Bulk 3 are composed of plants with different levels of resistance (asymptomatic, long symptom delay and mild symptom delay, respectively) but homozygous for the resistant allele at the CPMSNP315 marker, while Bulk 4 and Bulk 5 are composed of plants with severe ToLCNDV symptoms and PS 'Piñonet' homozygous at the CPMSNP315 position.
Results are shown for two QTL detection methods, the G-prime analysis method and the QTL-seq analysis method. G-prime: -log 10 (p-value), derived from the G' value. The horizontal red line indicate the 5% genome-wide false discovery rate threshold. QTL-seq : Distribution of ΔSNP-index calculated in a 1 Mb sliding window using a tricube-smoothing kernel. Red lines and blue lines indicate 95% and 99% confidence intervals, respectively.
| | 1 vs 2 | 1 vs 3 |
| --- | --- | --- |
| G-prime | | |
| QTL-seq | | |
| | 1 vs 4 | 1 vs 5 |
| G-prime | | |
| QTL-seq | | |
| | 2 vs 3 | 2 vs 4 |
| G-prime | | |
| QTL-seq | | |
| | 2 vs 5 | 3 vs 4 |
| G-prime | | |
| QTL-seq | | |
| | 3 vs 5 | 4 vs 5 |
| G-prime | | |
| QTL-seq | | |

## Slide 2
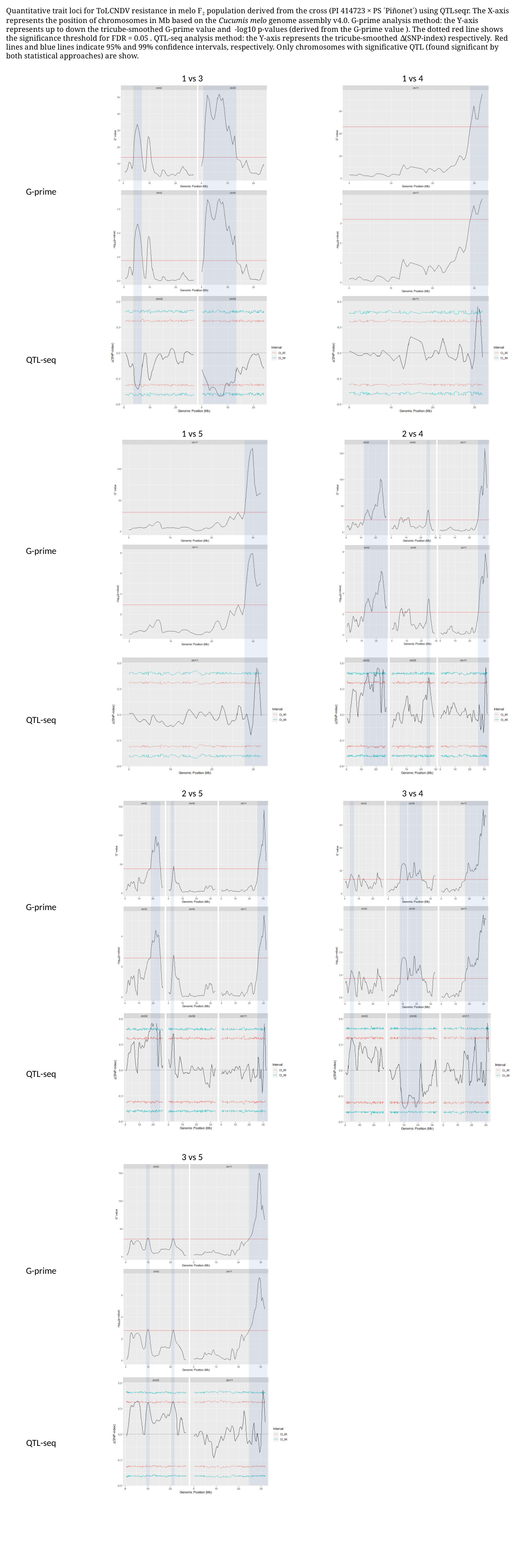

Quantitative trait loci for ToLCNDV resistance in melo F2 population derived from the cross (PI 414723 × PS ´Piñonet´) using QTLseqr. The X-axis represents the position of chromosomes in Mb based on the Cucumis melo genome assembly v4.0. G-prime analysis method: the Y-axis represents up to down the tricube-smoothed G-prime value and -log10 p-values (derived from the G-prime value ). The dotted red line shows the significance threshold for FDR = 0.05 . QTL-seq analysis method: the Y-axis represents the tricube-smoothed ∆(SNP-index) respectively. Red lines and blue lines indicate 95% and 99% confidence intervals, respectively. Only chromosomes with significative QTL (found significant by both statistical approaches) are show.
| | 1 vs 3 | 1 vs 4 |
| --- | --- | --- |
| G-prime | | |
| | | |
| QTL-seq | | |
| | 1 vs 5 | 2 vs 4 |
| G-prime | | |
| | | |
| QTL-seq | | |
| | 2 vs 5 | 3 vs 4 |
| G-prime | | |
| | | |
| QTL-seq | | |
| | 3 vs 5 | |
| G-prime | | |
| | | |
| QTL-seq | | |
| | | |
