# Supplementary material for: Genetic Dissection of ToLCNDV Resistance in Resistant Sources of Cucumis melo
Source: Int J Mol Sci. 2024 Aug 15;25(16):8880. doi: 10.3390/ijms25168880 (PMC11354858; doi:10.3390/ijms25168880)
Supplement: Supplementary file 1 [file ijms-25-08880-s001.zip › Figure S4.pptx]

## Slide 1
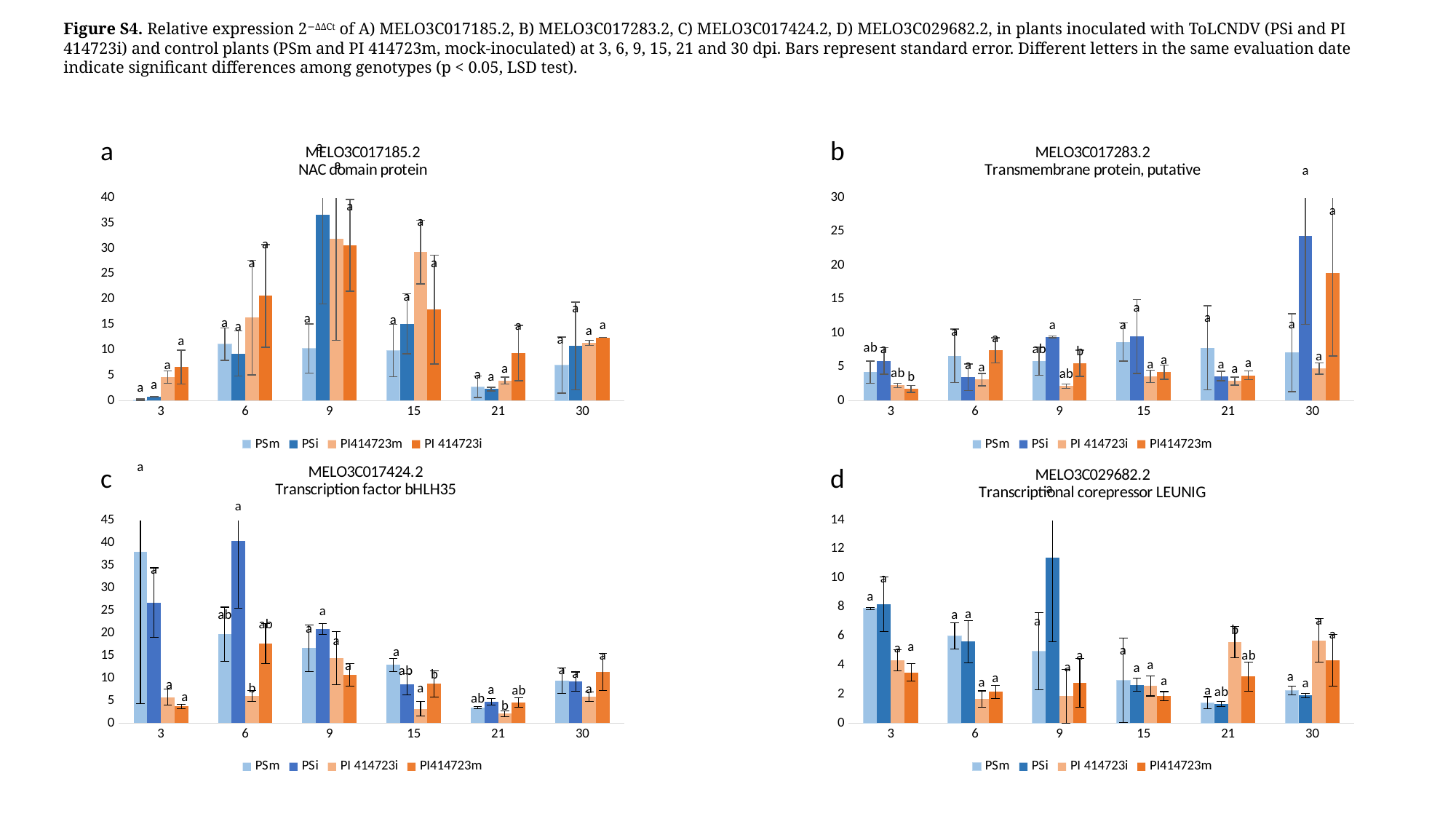

Figure S4. Relative expression 2−ΔΔCt of A) MELO3C017185.2, B) MELO3C017283.2, C) MELO3C017424.2, D) MELO3C029682.2, in plants inoculated with ToLCNDV (PSi and PI 414723i) and control plants (PSm and PI 414723m, mock-inoculated) at 3, 6, 9, 15, 21 and 30 dpi. Bars represent standard error. Different letters in the same evaluation date indicate significant differences among genotypes (p < 0.05, LSD test).
### Chart: MELO3C017185.2
NAC domain protein
| Category | PSm | PSi | PI414723m | PI 414723i |
|---|---|---|---|---|
| 3 | 0.187672686 | 0.7249656276666667 | 4.638815108666667 | 6.616765289 |
| 6 | 11.139433762666668 | 9.300054613 | 16.347551580666664 | 20.645012464 |
| 9 | 10.281128781333335 | 36.638543495 | 31.860535398499998 | 30.58792966 |
| 15 | 9.886592384333333 | 15.168372389333333 | 29.272484600000002 | 17.917682127666666 |
| 21 | 2.7182013166666668 | 2.300490820333333 | 3.963061249666666 | 9.3899772495 |
| 30 | 7.015991815333334 | 10.78415275 | 11.395848976666665 | 12.45985795 |a
### Chart: MELO3C017283.2
Transmembrane protein, putative
| Category | PSm | PSi | PI 414723i | PI414723m |
|---|---|---|---|---|
| 3 | 4.235176821207633 | 5.897652539894252 | 2.2491847706316093 | 1.7294678493399465 |
| 6 | 6.650123730771521 | 3.45226972654239 | 3.1153389043143984 | 7.4392766086154545 |
| 9 | 5.830499889809922 | 9.432277318000727 | 2.1472268552561458 | 5.527978525574823 |
| 15 | 8.679680220259117 | 9.494152187083222 | 3.609484167755929 | 4.197786666605993 |
| 21 | 7.805001368190016 | 3.6382896560197673 | 2.901246025250444 | 3.749123377576174 |
| 30 | 7.089134535122085 | 24.318873908945708 | 4.759521460617499 | 18.86849095477515 |b
### Chart: MELO3C017424.2
Transcription factor bHLH35
| Category | PSm | PSi | PI 414723i | PI414723m |
|---|---|---|---|---|
| 3 | 37.98563320720076 | 26.708394886580226 | 5.762490863356899 | 3.708656941928824 |
| 6 | 19.71619331817116 | 40.34464189738496 | 6.008438092785684 | 17.65209502340228 |
| 9 | 16.60588650996924 | 20.915843111719965 | 14.416804922380859 | 10.734603407080861 |
| 15 | 12.934443935883905 | 8.574873521807351 | 3.172813208848584 | 8.707132766288181 |
| 21 | 3.425335392381199 | 4.7584085118738555 | 2.0780518042706304 | 4.6081797295932745 |
| 30 | 9.427324393619404 | 9.182151070791448 | 5.887052557307352 | 11.423360970436276 |
### Chart: MELO3C029682.2
Transcriptional corepressor LEUNIG
| Category | PSm | PSi | PI 414723i | PI414723m |
|---|---|---|---|---|
| 3 | 7.908539607760128 | 8.214014484755976 | 4.331023141360904 | 3.4890098502550675 |
| 6 | 6.017454188411019 | 5.621503629788208 | 1.6656700116670657 | 2.152889051411222 |
| 9 | 4.9495548986463875 | 11.439244632410832 | 1.8530249859844747 | 2.7899928565755525 |
| 15 | 2.947446187602581 | 2.647673242712417 | 2.5569705710849333 | 1.8670793745504026 |
| 21 | 1.4022244658263168 | 1.3408267695637772 | 5.597227081491069 | 3.2212036957410675 |
| 30 | 2.2652988346760345 | 1.9228339743911695 | 5.713966265086962 | 4.333040094670821 |c
d
