# Supplementary material for: Genetic Dissection of ToLCNDV Resistance in Resistant Sources of Cucumis melo
Source: Int J Mol Sci. 2024 Aug 15;25(16):8880. doi: 10.3390/ijms25168880 (PMC11354858; doi:10.3390/ijms25168880)
Supplement: Supplementary file 1 [file ijms-25-08880-s001.zip › Figure S5.pptx]

## Slide 1
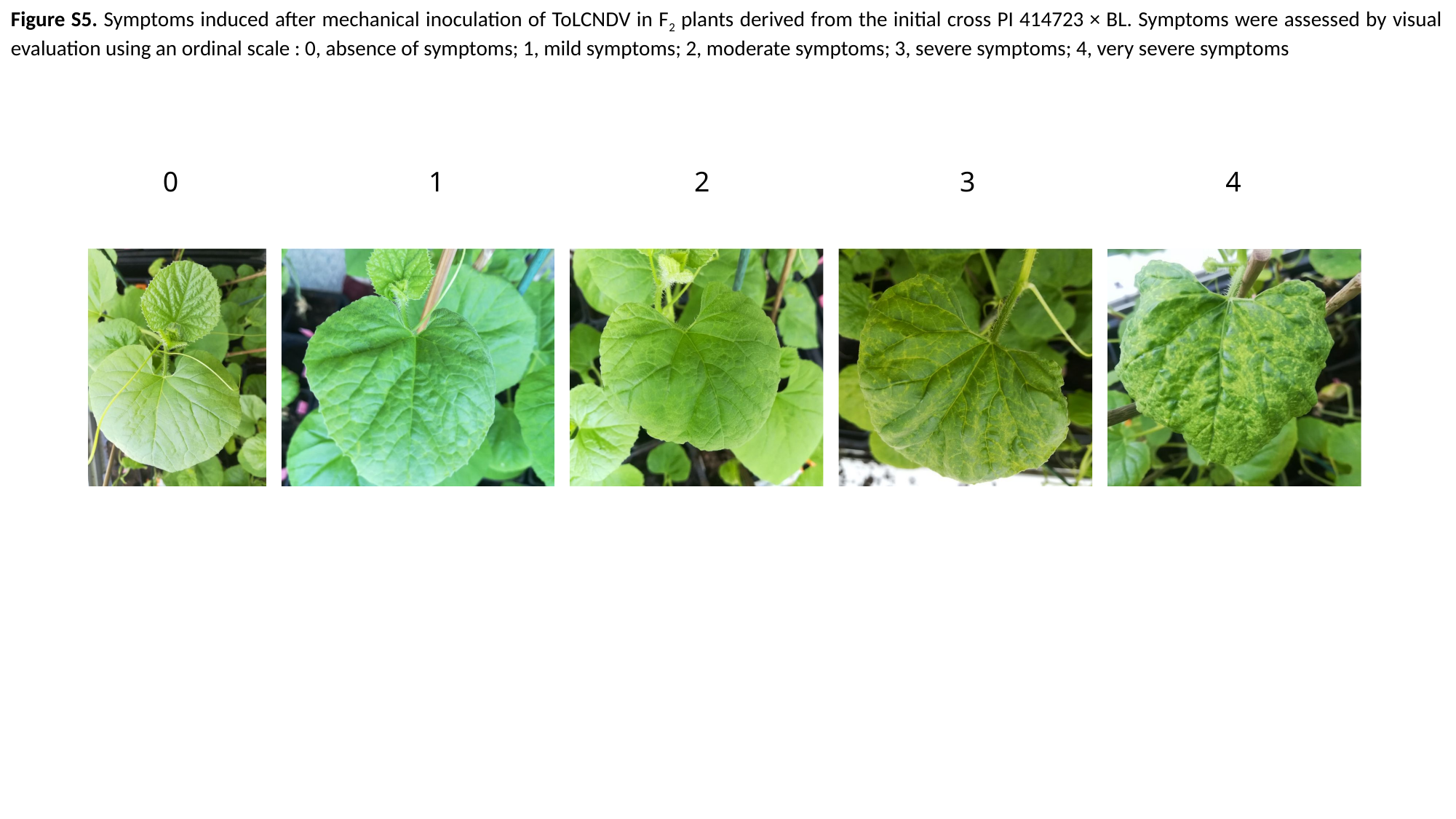

Figure S5. Symptoms induced after mechanical inoculation of ToLCNDV in F2 plants derived from the initial cross PI 414723 × BL. Symptoms were assessed by visual evaluation using an ordinal scale : 0, absence of symptoms; 1, mild symptoms; 2, moderate symptoms; 3, severe symptoms; 4, very severe symptoms
0
1
2
3
4
